# Supplementary material for: Mapping brain growth and sex differences across prenatal to postnatal development
Source: Sci Rep. 2026 Jan 15;16:3843. doi: 10.1038/s41598-025-33981-w (PMC12852117; doi:10.1038/s41598-025-33981-w)

Supplementary Materials: Mapping Brain Growth and Sex Differences Across Prenatal to Postnatal Development

Yumnah T. Khan, Alex Tsompanidis, Marcin A. Radecki, Carrie Allison, Meng-Chuan Lai, Richard A. I. Bethlehem, and Simon Baron-Cohen

Supplementary Table 1: p2-17

Supplementary Table 2: p18-34

Supplementary Table 3: p35-40

Supplementary Table 4: p41-46

Supplementary Table 5: p47-62

Supplementary Table 6: p63

Supplementary Figure 1: p64

Supplementary Figure 2: p65

Supplementary Figure 3: p65-66

Supplementary Figure 4: p66

Supplementary Table 1 - Absolute Analysis

Region	Best fitting model	Term	B	Std error	P value (FDR adjusted)	Standardised B	Std Error (std beta)
Total Brain Volume	Quadratic	Gestational age at scan	17995.02	269.52	<0.001	0.18	0.02
		Sex	19337.23	2919.29	<0.001	0.08	0.01
		Gestational age at scan^2	179.47	34.54	<0.001	0.10	0.02
		Gestational age at scan*sex	1764.56	387.00	<0.001	0.02	0.02
		Gestational age at scan^2*sex	35.40	50.01	0.534	0.82	0.02
Total Gray Matter	Quadratic	Gestational age at scan	12641.40	155.86	<0.001	1.07	0.01
		Sex	8261.37	1675.74	<0.001	0.14	0.01
		Gestational age at scan^2	276.71	19.91	<0.001	0.18	0.01
		Gestational age at scan*sex	1215.97	224.23	<0.001	0.10	0.02
		Gestational age at scan^2*sex	47.68	28.82	0.138	0.03	0.02
Total White Matter	Quadratic	Gestational age at scan	5345.31	125.46	<0.001	0.82	0.02
		Sex	10954.07	1356.43	<0.001	0.25	0.02
		Gestational age at scan^2	-97.90	16.09	<0.001	-0.12	0.02
		Gestational age at scan*sex	556.38	178.86	<0.001	0.09	0.03
		Gestational age at scan^2*sex	-9.12	23.15	0.717	-0.01	0.03
CSF	Cubic	Gestational age at scan	-1078.45	174.77	<0.001	-0.40	0.06
		Sex	-1270.66	1714.16	0.592	0.17	0.05
		Gestational age at scan^2	142.32	37.18	<0.001	0.40	0.11
		Gestational age at scan^3	30.41	2.79	<0.001	1.30	0.12
		Gestational age at scan*sex	-83.01	240.22	0.844	-0.03	0.09
		Gestational age at scan^2*sex	181.88	51.21	0.001	0.52	0.15
		Gestational age at scan^3*sex	13.11	3.90	0.002	0.56	0.17
Total Intracranial Volume	Cubic	Gestational age at scan	16891.11	422.02	<0.001	0.86	0.02
		Sex	17361.92	4576.39	<0.001	0.19	0.02
		Gestational age at scan^2	317.30	95.53	0.002	0.13	0.04
		Gestational age at scan^3	30.33	6.95	<0.001	0.18	0.04
		Gestational age at scan*sex	1620.52	599.25	0.013	0.08	0.03
		Gestational age at scan^2*sex	242.93	134.38	0.106	0.10	0.05
		Gestational age at scan^3*sex	15.08	10.02	0.187	0.09	0.06
Hippocampus left	Linear	Gestational age at scan	32.15	0.60	<0.001	0.87	0.02

		Sex	35.26	6.24	<0.001	0.15	0.03
		Gestational age at scan*sex	4.30	0.84	<0.001	0.12	0.02
Hippocampus right	Linear	Gestational age at scan	36.11	0.61	<0.001	0.88	0.01
		Sex	35.95	6.34	<0.001	0.14	0.02
		Gestational age at scan*sex	5.21	0.84	<0.001	0.13	0.02
Amygdala left	Cubic	Gestational age at scan	-0.05	0.01	<0.001	0.97	0.03
		Sex	29.26	7.51	<0.001	0.15	0.02
		Gestational age at scan^2	-0.51	0.17	0.007	-0.15	0.05
		Gestational age at scan^3	2.31	1.19	0.095	-0.21	0.06
		Gestational age at scan*sex	-0.19	0.24	0.544	0.09	0.04
		Gestational age at scan^2*sex	-0.01	0.02	0.551	-0.05	0.07
		Gestational age at scan^3*sex	25.95	0.85	<0.001	-0.06	0.08
Amygdala right	Cubic	Gestational age at scan	-0.04	0.01	0.013	0.97	0.03
		Sex	32.75	8.68	<0.001	0.14	0.02
		Gestational age at scan^2	-0.35	0.19	0.120	-0.09	0.05
		Gestational age at scan^3	2.88	1.33	0.061	-0.16	0.05
		Gestational age at scan*sex	-0.28	0.27	0.403	0.09	0.04
		Gestational age at scan^2*sex	-0.02	0.02	0.396	-0.07	0.07
		Gestational age at scan^3*sex	29.41	0.95	<0.001	-0.09	0.08
Anterior temporal lobe medial part left GM	Quadratic	Gestational age at scan	48.81	1.20	<0.001	0.99	0.02
		Sex	48.63	12.97	<0.001	0.18	0.03
		Gestational age at scan^2	0.83	0.15	<0.001	0.13	0.02
		Gestational age at scan*sex	5.27	1.81	0.007	0.11	0.04
		Gestational age at scan^2*sex	0.22	0.23	0.401	0.03	0.04
Anterior temporal lobe medial part right GM	Quadratic	Gestational age at scan	52.84	1.16	<0.001	1.02	0.02
		Sex	58.71	13.03	<0.001	0.17	0.03
		Gestational age at scan^2	1.04	0.15	<0.001	0.15	0.02
		Gestational age at scan*sex	3.32	1.80	0.089	0.06	0.03
		Gestational age at scan^2*sex	-0.07	0.23	0.794	-0.01	0.03
Anterior temporal lobe lateral part left GM	Quadratic	Gestational age at scan	56.41	1.42	<0.001	1.01	0.03
		Sex	60.29	14.65	<0.001	0.19	0.03

		Gestational age at scan^2	1.21	0.18	<0.001	0.17	0.03
		Gestational age at scan*sex	5.69	1.98	0.007	0.10	0.04
		Gestational age at scan^2*sex	0.13	0.25	0.665	0.02	0.04
Anterior temporal lobe lateral part right GM	Quadratic	Gestational age at scan	62.72	1.34	<0.001	1.08	0.02
		Sex	53.87	13.93	<0.001	0.13	0.02
		Gestational age at scan^2	1.64	0.17	<0.001	0.22	0.02
		Gestational age at scan*sex	2.53	1.91	0.227	0.04	0.03
		Gestational age at scan^2*sex	-0.13	0.24	0.660	-0.02	0.03
Gyri parahippocampalis et ambiens anterior part left GM	Cubic	Gestational age at scan	57.22	1.64	<0.001	0.98	0.03
		Sex	-0.08	0.03	0.010	0.14	0.02
		Gestational age at scan^2	72.85	17.97	<0.001	-0.06	0.05
		Gestational age at scan^3	-0.48	0.36	0.273	-0.15	0.05
		Gestational age at scan*sex	4.07	2.30	0.130	0.07	0.04
		Gestational age at scan^2*sex	-0.76	0.51	0.219	-0.10	0.07
		Gestational age at scan^3*sex	-0.05	0.04	0.297	-0.10	0.07
Gyri parahippocampalis et ambiens anterior part right GM	Cubic	Gestational age at scan	47.66	16.59	0.009	1.01	0.03
		Sex	0.17	0.34	0.717	0.10	0.02
		Gestational age at scan^2	-0.05	0.02	0.072	0.02	0.05
		Gestational age at scan^3	4.92	2.11	0.043	-0.11	0.05
		Gestational age at scan*sex	-0.55	0.47	0.339	0.09	0.04
		Gestational age at scan^2*sex	-0.04	0.03	0.288	-0.08	0.07
		Gestational age at scan^3*sex	54.92	1.50	<0.001	-0.09	0.07
Superior temporal gyrus middle part left GM	Quadratic	Gestational age at scan	186.63	3.22	<0.001	1.05	0.02
		Sex	140.19	33.50	<0.001	0.15	0.02
		Gestational age at scan^2	4.26	0.41	<0.001	0.19	0.02
		Gestational age at scan*sex	20.07	4.61	<0.001	0.11	0.03
		Gestational age at scan^2*sex	0.63	0.58	0.327	0.03	0.03
Superior temporal gyrus middle part right GM	Quadratic	Gestational age at scan	189.68	3.07	<0.001	1.06	0.02
		Sex	141.32	32.81	<0.001	0.14	0.02
		Gestational age at scan^2	4.41	0.39	<0.001	0.19	0.02
		Gestational age at scan*sex	16.51	4.43	0.001	0.09	0.02

		Gestational age at scan^2*sex	0.39	0.56	0.535	0.02	0.02
Medial and inferior temporal gyri anterior part left GM	Quadratic	Gestational age at scan	156.92	3.34	<0.001	1.08	0.02
		Sex	107.16	34.10	0.003	0.16	0.02
		Gestational age at scan^2	4.81	0.43	<0.001	0.26	0.02
		Gestational age at scan*sex	19.04	4.69	<0.001	0.13	0.03
		Gestational age at scan^2*sex	0.99	0.60	0.128	0.05	0.03
Medial and inferior temporal gyri anterior part right GM	Quadratic	Gestational age at scan	173.87	3.19	<0.001	1.10	0.02
		Sex	84.26	33.65	0.018	0.14	0.02
		Gestational age at scan^2	5.35	0.41	<0.001	0.26	0.02
		Gestational age at scan*sex	21.24	4.64	<0.001	0.13	0.03
		Gestational age at scan^2*sex	1.30	0.59	0.039	0.06	0.03
Lateral occipitotemporal gyrus fusiformis anterior part left GM	Quadratic	Gestational age at scan	40.46	1.21	<0.001	0.96	0.03
		Sex	33.57	12.00	0.008	0.14	0.03
		Gestational age at scan^2	0.52	0.15	0.001	0.10	0.03
		Gestational age at scan*sex	3.33	1.65	0.061	0.08	0.04
		Gestational age at scan^2*sex	0.08	0.21	0.743	0.01	0.04
Lateral occipitotemporal gyrus fusiformis anterior part right GM	Quadratic	Gestational age at scan	45.19	1.28	<0.001	0.98	0.03
		Sex	30.32	12.95	0.027	0.13	0.03
		Gestational age at scan^2	0.81	0.16	<0.001	0.14	0.03
		Gestational age at scan*sex	5.20	1.79	0.007	0.11	0.04
		Gestational age at scan^2*sex	0.17	0.23	0.501	0.03	0.04
Cerebellum left	Quadratic	Gestational age at scan	897.11	10.74	<0.001	1.12	0.01
		Sex	378.22	115.65	0.002	0.09	0.01
		Gestational age at scan^2	24.30	1.37	<0.001	0.23	0.01
		Gestational age at scan*sex	53.13	16.02	0.002	0.07	0.02
		Gestational age at scan^2*sex	1.74	2.05	0.451	0.02	0.02
Cerebellum right	Quadratic	Gestational age at scan	843.72	10.36	<0.001	1.10	0.01
		Sex	367.09	114.18	0.002	0.10	0.01
		Gestational age at scan^2	21.01	1.32	<0.001	0.21	0.01
		Gestational age at scan*sex	61.78	15.79	<0.001	0.08	0.02
		Gestational age at scan^2*sex	2.43	2.02	0.276	0.02	0.02

Brainstem	Quadratic	Gestational age at scan	228.87	4.60	<0.001	0.90	0.02
		Sex	178.39	48.36	<0.001	0.13	0.02
		Gestational age at scan^2	-1.96	0.59	0.002	-0.06	0.02
		Gestational age at scan*sex	18.75	6.46	0.007	0.07	0.03
		Gestational age at scan^2*sex	0.88	0.83	0.340	0.03	0.03
Insula right GM	Quadratic	Gestational age at scan	97.18	1.73	<0.001	1.00	0.02
		Sex	102.69	18.72	<0.001	0.17	0.02
		Gestational age at scan^2	1.41	0.22	<0.001	0.11	0.02
		Gestational age at scan*sex	9.37	2.50	0.001	0.10	0.03
		Gestational age at scan^2*sex	0.04	0.32	0.914	0.00	0.03
Insula left GM	Quadratic	Gestational age at scan	104.60	1.85	<0.001	0.98	0.02
		Sex	94.92	20.29	<0.001	0.17	0.02
		Gestational age at scan^2	1.26	0.24	<0.001	0.09	0.02
		Gestational age at scan*sex	14.00	2.73	<0.001	0.13	0.03
		Gestational age at scan^2*sex	0.49	0.35	0.206	0.04	0.03
Occipital lobe right GM	Quadratic	Gestational age at scan	736.15	12.89	<0.001	1.06	0.02
		Sex	389.09	133.82	0.006	0.12	0.02
		Gestational age at scan^2	15.69	1.65	<0.001	0.17	0.02
		Gestational age at scan*sex	78.63	18.42	<0.001	0.11	0.03
		Gestational age at scan^2*sex	3.95	2.38	0.126	0.04	0.03
Occipital lobe left GM	Quadratic	Gestational age at scan	772.20	12.18	<0.001	1.09	0.02
		Sex	471.98	129.38	<0.001	0.12	0.02
		Gestational age at scan^2	18.81	1.56	<0.001	0.20	0.02
		Gestational age at scan*sex	58.41	17.60	0.002	0.08	0.02
		Gestational age at scan^2*sex	1.73	2.27	0.499	0.02	0.02
Gyri parahippocampalis et ambiens posterior part right GM	Quadratic	Gestational age at scan	49.23	1.03	<0.001	1.04	0.02
		Sex	30.93	11.12	0.008	0.11	0.02
		Gestational age at scan^2	1.01	0.13	<0.001	0.17	0.02
		Gestational age at scan*sex	2.65	1.53	0.110	0.06	0.03
		Gestational age at scan^2*sex	0.02	0.19	0.914	0.00	0.03
Gyri parahippocampalis et ambiens posterior part left GM	Quadratic	Gestational age at scan	46.28	1.08	<0.001	1.01	0.02

		Sex	19.56	11.17	0.103	0.07	0.02
		Gestational age at scan^2	0.73	0.14	<0.001	0.12	0.02
		Gestational age at scan*sex	2.01	1.53	0.233	0.04	0.03
		Gestational age at scan^2*sex	0.02	0.19	0.922	0.00	0.03
Lateral occipitotemporal gyrus fusiformis posterior part right GM	Quadratic	Gestational age at scan	73.47	1.54	<0.001	1.11	0.02
		Sex	34.65	15.36	0.033	0.10	0.02
		Gestational age at scan^2	2.30	0.19	<0.001	0.27	0.02
		Gestational age at scan*sex	5.41	2.13	0.018	0.08	0.03
		Gestational age at scan^2*sex	0.24	0.27	0.435	0.03	0.03
Lateral occipitotemporal gyrus fusiformis posterior part left GM	Quadratic	Gestational age at scan	66.61	1.51	<0.001	1.07	0.02
		Sex	35.90	15.67	0.031	0.11	0.02
		Gestational age at scan^2	1.86	0.19	<0.001	0.23	0.02
		Gestational age at scan*sex	4.96	2.12	0.029	0.08	0.03
		Gestational age at scan^2*sex	0.20	0.26	0.501	0.02	0.03
Medial and inferior temporal gyri posterior part right GM	Quadratic	Gestational age at scan	256.02	4.34	<0.001	1.10	0.02
		Sex	270.53	45.32	<0.001	0.19	0.02
		Gestational age at scan^2	7.80	0.56	<0.001	0.26	0.02
		Gestational age at scan*sex	25.33	6.27	<0.001	0.11	0.03
		Gestational age at scan^2*sex	0.36	0.81	0.707	0.01	0.03
Medial and inferior temporal gyri posterior part left GM	Quadratic	Gestational age at scan	263.98	4.53	<0.001	1.09	0.02
		Sex	212.00	47.87	<0.001	0.18	0.02
		Gestational age at scan^2	8.26	0.58	<0.001	0.26	0.02
		Gestational age at scan*sex	33.67	6.57	<0.001	0.14	0.03
		Gestational age at scan^2*sex	1.57	0.84	0.084	0.05	0.03
Superior temporal gyrus posterior part right GM	Quadratic	Gestational age at scan	82.47	1.63	<0.001	1.04	0.02
		Sex	30.87	17.59	0.103	0.12	0.02
		Gestational age at scan^2	1.90	0.21	<0.001	0.19	0.02
		Gestational age at scan*sex	11.47	2.39	<0.001	0.14	0.03
		Gestational age at scan^2*sex	0.79	0.30	0.015	0.08	0.03
Superior temporal gyrus posterior part left GM	Quadratic	Gestational age at scan	72.26	1.56	<0.001	1.02	0.02
		Sex	19.01	16.06	0.280	0.11	0.02

		Gestational age at scan^2	1.44	0.20	<0.001	0.16	0.02
		Gestational age at scan*sex	10.71	2.27	<0.001	0.15	0.03
		Gestational age at scan^2*sex	0.79	0.29	0.012	0.09	0.03
Cingulate gyrus anterior part right GM	Quadratic	Gestational age at scan	84.38	2.05	<0.001	0.99	0.02
		Sex	90.38	21.97	<0.001	0.18	0.03
		Gestational age at scan^2	1.65	0.26	<0.001	0.15	0.02
		Gestational age at scan*sex	8.42	2.94	0.007	0.10	0.03
		Gestational age at scan^2*sex	0.13	0.37	0.775	0.01	0.03
Cingulate gyrus anterior part left GM	Cubic	Gestational age at scan	106.48	28.32	0.001	0.96	0.04
		Sex	0.46	0.59	0.559	0.12	0.03
		Gestational age at scan^2	-0.04	0.04	0.525	0.05	0.06
		Gestational age at scan^3	6.40	3.86	0.160	-0.06	0.07
		Gestational age at scan*sex	-2.05	0.84	0.033	0.08	0.05
		Gestational age at scan^2*sex	-0.15	0.06	0.044	-0.21	0.09
		Gestational age at scan^3*sex	72.14	2.73	<0.001	-0.23	0.10
Cingulate gyrus posterior part right GM	Quadratic	Gestational age at scan	100.09	2.33	<0.001	0.99	0.02
		Sex	83.58	24.52	0.001	0.18	0.02
		Gestational age at scan^2	1.95	0.29	<0.001	0.15	0.02
		Gestational age at scan*sex	15.56	3.30	<0.001	0.15	0.03
		Gestational age at scan^2*sex	0.84	0.42	0.062	0.06	0.03
Cingulate gyrus posterior part left GM	Quadratic	Gestational age at scan	100.92	2.20	<0.001	1.01	0.02
		Sex	82.09	23.05	0.001	0.16	0.02
		Gestational age at scan^2	2.02	0.28	<0.001	0.16	0.02
		Gestational age at scan*sex	12.44	3.12	<0.001	0.12	0.03
		Gestational age at scan^2*sex	0.51	0.40	0.246	0.04	0.03
Frontal lobe right GM	Quadratic	Gestational age at scan	1536.84	23.22	<0.001	1.05	0.02
		Sex	1221.64	250.03	<0.001	0.17	0.02
		Gestational age at scan^2	31.56	2.97	<0.001	0.17	0.02
		Gestational age at scan*sex	189.90	33.36	<0.001	0.13	0.02
		Gestational age at scan^2*sex	8.07	4.30	0.082	0.04	0.02
Frontal lobe left GM	Quadratic	Gestational age at scan	1544.31	23.22	<0.001	1.05	0.02

		Sex	1178.32	248.66	<0.001	0.16	0.02
		Gestational age at scan^2	32.90	2.97	<0.001	0.17	0.02
		Gestational age at scan*sex	174.56	33.22	<0.001	0.12	0.02
		Gestational age at scan^2*sex	6.82	4.29	0.143	0.04	0.02
Parietal lobe right GM	Quadratic	Gestational age at scan	1181.49	15.98	<0.001	1.09	0.01
		Sex	574.74	169.10	0.001	0.11	0.02
		Gestational age at scan^2	28.96	2.05	<0.001	0.21	0.01
		Gestational age at scan*sex	103.89	22.80	<0.001	0.10	0.02
		Gestational age at scan^2*sex	5.11	2.94	0.109	0.04	0.02
Parietal lobe left GM	Quadratic	Gestational age at scan	1188.28	16.14	<0.001	1.09	0.01
		Sex	558.93	171.25	0.002	0.12	0.02
		Gestational age at scan^2	29.28	2.07	<0.001	0.21	0.01
		Gestational age at scan*sex	121.66	23.17	<0.001	0.11	0.02
		Gestational age at scan^2*sex	6.62	2.98	0.039	0.05	0.02
Temporal lobe right GM (merged region)	Quadratic	Gestational age at scan	1038.88	13.99	<0.001	1.09	0.01
		Sex	780.00	151.32	<0.001	0.15	0.02
		Gestational age at scan^2	26.99	1.78	<0.001	0.22	0.01
		Gestational age at scan*sex	97.02	20.38	<0.001	0.10	0.02
		Gestational age at scan^2*sex	2.87	2.61	0.319	0.02	0.02
Temporal lobe left GM (merged region)	Quadratic	Gestational age at scan	729.97	10.85	<0.001	1.06	0.02
		Sex	542.04	114.07	<0.001	0.15	0.02
		Gestational age at scan^2	16.20	1.38	<0.001	0.18	0.02
		Gestational age at scan*sex	72.41	15.53	<0.001	0.11	0.02
		Gestational age at scan^2*sex	2.60	1.99	0.233	0.03	0.02
Caudate nucleus right	Cubic	Gestational age at scan	112.81	2.41	<0.001	1.06	0.02
		Sex	-0.18	0.04	<0.001	0.14	0.02
		Gestational age at scan^2	108.20	29.20	<0.001	-0.05	0.04
		Gestational age at scan^3	-0.63	0.57	0.365	-0.19	0.04
		Gestational age at scan*sex	4.56	3.38	0.265	0.04	0.03
		Gestational age at scan^2*sex	-0.51	0.80	0.642	-0.04	0.06
		Gestational age at scan^3*sex	-0.04	0.06	0.629	-0.04	0.06

Caudate nucleus left	Cubic	Gestational age at scan	110.24	2.38	<0.001	1.06	0.02
		Sex	-0.16	0.04	<0.001	0.13	0.02
		Gestational age at scan^2	94.09	29.59	0.004	-0.02	0.04
		Gestational age at scan^3	-0.30	0.57	0.717	-0.17	0.05
		Gestational age at scan*sex	3.71	3.34	0.365	0.04	0.03
		Gestational age at scan^2*sex	-0.22	0.81	0.866	-0.02	0.06
		Gestational age at scan^3*sex	-0.01	0.06	0.873	-0.02	0.06
Thalamus right	Cubic	Gestational age at scan	247.03	3.65	<0.001	1.12	0.02
		Sex	-0.50	0.06	<0.001	0.11	0.01
		Gestational age at scan^2	113.70	39.29	0.009	-0.07	0.03
		Gestational age at scan^3	-1.91	0.80	0.039	-0.26	0.03
		Gestational age at scan*sex	18.71	5.10	0.001	0.09	0.02
		Gestational age at scan^2*sex	0.68	1.13	0.664	0.02	0.04
		Gestational age at scan^3*sex	-0.04	0.08	0.727	-0.02	0.04
Thalamus left	Cubic	Gestational age at scan	241.04	3.61	<0.001	1.13	0.02
		Sex	-0.48	0.06	<0.001	0.12	0.01
		Gestational age at scan^2	127.10	37.46	0.002	-0.06	0.03
		Gestational age at scan^3	-1.58	0.78	0.083	-0.26	0.03
		Gestational age at scan*sex	15.83	5.08	0.006	0.07	0.02
		Gestational age at scan^2*sex	0.72	1.10	0.630	0.03	0.04
		Gestational age at scan^3*sex	-0.02	0.08	0.856	-0.01	0.05
Subthalamic nucleus right	Cubic	Gestational age at scan	0.05	0.01	<0.001	0.26	0.05
		Sex	6.62	3.36	0.087	0.21	0.03
		Gestational age at scan^2	0.20	0.07	0.017	0.18	0.07
		Gestational age at scan^3	0.89	0.53	0.151	0.76	0.08
		Gestational age at scan*sex	0.19	0.10	0.128	0.11	0.06
		Gestational age at scan^2*sex	0.01	0.01	0.224	0.17	0.10
		Gestational age at scan^3*sex	2.15	0.38	<0.001	0.17	0.12
Subthalamic nucleus left	Cubic	Gestational age at scan	0.05	0.01	<0.001	-0.07	0.06
		Sex	4.65	3.35	0.246	0.18	0.04
		Gestational age at scan^2	0.14	0.07	0.095	0.17	0.09

		Gestational age at scan^3	1.05	0.51	0.078	0.95	0.10
		Gestational age at scan*sex	0.10	0.10	0.449	0.16	0.08
		Gestational age at scan^2*sex	0.00	0.01	0.685	0.12	0.12
		Gestational age at scan^3*sex	-0.44	0.36	0.320	0.08	0.14
Lentiform nucleus right	Quadratic	Gestational age at scan	155.90	3.32	<0.001	1.00	0.02
		Sex	118.98	33.73	0.001	0.14	0.02
		Gestational age at scan^2	2.41	0.41	<0.001	0.12	0.02
		Gestational age at scan*sex	15.82	4.51	0.001	0.10	0.03
		Gestational age at scan^2*sex	0.41	0.56	0.517	0.02	0.03
Lentiform nucleus left	Cubic	Gestational age at scan	165.27	3.53	<0.001	1.00	0.02
		Sex	155.77	42.17	0.001	0.15	0.02
		Gestational age at scan^2	0.50	0.83	0.660	0.03	0.04
		Gestational age at scan^3	-0.10	0.06	0.127	-0.07	0.04
		Gestational age at scan*sex	19.87	4.99	<0.001	0.12	0.03
		Gestational age at scan^2*sex	-0.57	1.18	0.738	-0.03	0.06
		Gestational age at scan^3*sex	-0.10	0.08	0.352	-0.07	0.06
Corpus Callosum	Quadratic	Gestational age at scan	165.71	3.95	<0.001	1.06	0.03
		Sex	115.18	40.45	0.007	0.15	0.02
		Gestational age at scan^2	4.62	0.50	<0.001	0.23	0.02
		Gestational age at scan*sex	13.81	5.45	0.018	0.09	0.03
		Gestational age at scan^2*sex	0.92	0.69	0.223	0.05	0.03
Lateral Ventricle left	Cubic	Gestational age at scan	0.48	0.19	0.028	-0.06	0.07
		Sex	-101.12	115.29	0.499	0.23	0.07
		Gestational age at scan^2	-0.81	2.51	0.840	-0.04	0.12
		Gestational age at scan^3	-5.53	16.26	0.830	0.35	0.14
		Gestational age at scan*sex	12.93	3.56	0.001	-0.03	0.10
		Gestational age at scan^2*sex	0.80	0.28	0.012	0.63	0.17
		Gestational age at scan^3*sex	-10.15	11.17	0.481	0.58	0.20
Lateral Ventricle right	Cubic	Gestational age at scan	0.38	0.16	0.034	-0.02	0.06
		Sex	63.21	87.34	0.582	0.33	0.07
		Gestational age at scan^2	-0.28	1.98	0.927	-0.02	0.11

		Gestational age at scan^3	-7.21	13.69	0.713	0.36	0.12
		Gestational age at scan*sex	8.20	2.80	0.009	-0.09	0.11
		Gestational age at scan^2*sex	0.57	0.23	0.028	0.57	0.17
		Gestational age at scan^3*sex	-2.39	9.52	0.873	0.62	0.21
Anterior temporal lobe medial part left WM	Quadratic	Gestational age at scan	36.13	1.18	<0.001	0.95	0.03
		Sex	25.56	12.08	0.046	0.15	0.03
		Gestational age at scan^2	0.67	0.15	<0.001	0.14	0.03
		Gestational age at scan*sex	4.44	1.65	0.012	0.12	0.04
		Gestational age at scan^2*sex	0.27	0.21	0.242	0.05	0.04
Anterior temporal lobe medial part right WM	Quadratic	Gestational age at scan	32.28	1.07	<0.001	0.92	0.03
		Sex	31.21	11.75	0.012	0.16	0.03
		Gestational age at scan^2	0.56	0.13	<0.001	0.12	0.03
		Gestational age at scan*sex	3.16	1.57	0.062	0.09	0.04
		Gestational age at scan^2*sex	0.10	0.19	0.660	0.02	0.04
Anterior temporal lobe lateral part left WM	Quadratic	Gestational age at scan	38.58	0.95	<0.001	1.03	0.03
		Sex	37.18	10.24	0.001	0.16	0.03
		Gestational age at scan^2	0.92	0.12	<0.001	0.19	0.03
		Gestational age at scan*sex	2.69	1.40	0.076	0.07	0.04
		Gestational age at scan^2*sex	0.02	0.18	0.921	0.00	0.04
Anterior temporal lobe lateral part right WM	Quadratic	Gestational age at scan	36.92	0.92	<0.001	1.04	0.03
		Sex	17.95	9.97	0.093	0.09	0.03
		Gestational age at scan^2	0.84	0.11	<0.001	0.18	0.02
		Gestational age at scan*sex	1.48	1.35	0.319	0.04	0.04
		Gestational age at scan^2*sex	0.04	0.17	0.838	0.01	0.04
Gyri parahippocampalis et ambiens anterior part left WM	Cubic	Gestational age at scan	-0.06	0.02	0.004	1.01	0.03
		Sex	20.71	12.02	0.139	0.13	0.03
		Gestational age at scan^2	-0.19	0.24	0.559	-0.04	0.05
		Gestational age at scan^3	3.22	1.53	0.070	-0.20	0.06
		Gestational age at scan*sex	0.16	0.34	0.740	0.10	0.05
		Gestational age at scan^2*sex	0.00	0.02	0.949	0.04	0.08
		Gestational age at scan^3*sex	33.34	1.09	<0.001	-0.01	0.09

Gyri parahippocampalis et ambiens anterior part right WM	Cubic	Gestational age at scan	-0.05	0.02	0.005	1.00	0.03
		Sex	25.98	10.58	0.029	0.15	0.03
		Gestational age at scan^2	-0.22	0.21	0.420	-0.06	0.05
		Gestational age at scan^3	2.81	1.36	0.076	-0.20	0.06
		Gestational age at scan*sex	-0.03	0.30	0.955	0.10	0.05
		Gestational age at scan^2*sex	-0.01	0.02	0.737	-0.01	0.08
		Gestational age at scan^3*sex	29.13	0.98	<0.001	-0.04	0.09
Superior temporal gyrus middle part left WM	Quadratic	Gestational age at scan	90.26	3.25	<0.001	0.76	0.03
		Sex	197.02	34.86	<0.001	0.21	0.03
		Gestational age at scan^2	-2.28	0.42	<0.001	-0.15	0.03
		Gestational age at scan*sex	2.23	4.71	0.685	0.02	0.04
		Gestational age at scan^2*sex	-0.89	0.60	0.179	-0.06	0.04
Superior temporal gyrus middle part right WM	Quadratic	Gestational age at scan	89.13	2.80	<0.001	0.80	0.03
		Sex	132.05	30.41	<0.001	0.18	0.03
		Gestational age at scan^2	-1.66	0.36	<0.001	-0.11	0.02
		Gestational age at scan*sex	6.71	4.09	0.131	0.06	0.04
		Gestational age at scan^2*sex	-0.11	0.52	0.856	-0.01	0.04
Medial and inferior temporal gyri anterior part left WM	Quadratic	Gestational age at scan	92.99	3.60	<0.001	0.74	0.03
		Sex	189.04	38.86	<0.001	0.22	0.03
		Gestational age at scan^2	-2.54	0.46	<0.001	-0.16	0.03
		Gestational age at scan*sex	7.33	5.32	0.209	0.06	0.04
		Gestational age at scan^2*sex	-0.34	0.69	0.673	-0.02	0.04
Medial and inferior temporal gyri anterior part right WM	Quadratic	Gestational age at scan	99.29	3.41	<0.001	0.74	0.03
		Sex	132.10	36.56	0.001	0.19	0.03
		Gestational age at scan^2	-2.90	0.44	<0.001	-0.17	0.03
		Gestational age at scan*sex	16.90	4.97	0.002	0.13	0.04
		Gestational age at scan^2*sex	0.66	0.64	0.351	0.04	0.04
Lateral occipitotemporal gyrus fusiformis anterior part left WM	Cubic	Gestational age at scan	-0.08	0.03	0.014	1.00	0.04
		Sex	51.53	17.32	0.007	0.21	0.03
		Gestational age at scan^2	-0.04	0.36	0.952	-0.01	0.07
		Gestational age at scan^3	5.54	2.38	0.043	-0.20	0.08

		Gestational age at scan*sex	0.05	0.51	0.956	0.13	0.06
		Gestational age at scan^2*sex	-0.01	0.04	0.824	0.01	0.09
		Gestational age at scan^3*sex	43.12	1.70	<0.001	-0.04	0.11
Lateral occipitotemporal gyrus fusiformis anterior part right WM	Linear	Gestational age at scan	34.07	0.83	<0.001	0.82	0.02
		Sex	65.14	8.82	<0.001	0.25	0.03
		Gestational age at scan*sex	4.87	1.16	<0.001	0.12	0.03
Insula right WM	Quadratic	Gestational age at scan	107.70	3.13	<0.001	0.81	0.02
		Sex	152.15	32.61	<0.001	0.20	0.02
		Gestational age at scan^2	-1.65	0.40	<0.001	-0.10	0.02
		Gestational age at scan*sex	16.16	4.43	0.001	0.12	0.03
		Gestational age at scan^2*sex	0.35	0.57	0.589	0.02	0.03
Insula left WM	Quadratic	Gestational age at scan	105.48	3.17	<0.001	0.80	0.02
		Sex	147.81	33.02	<0.001	0.18	0.02
		Gestational age at scan^2	-1.72	0.40	<0.001	-0.10	0.02
		Gestational age at scan*sex	14.54	4.47	0.002	0.11	0.03
		Gestational age at scan^2*sex	0.13	0.57	0.843	0.01	0.03
Occipital lobe right WM	Quadratic	Gestational age at scan	238.24	9.39	<0.001	0.72	0.03
		Sex	571.44	99.05	<0.001	0.28	0.03
		Gestational age at scan^2	-6.91	1.21	<0.001	-0.16	0.03
		Gestational age at scan*sex	43.63	13.30	0.002	0.13	0.04
		Gestational age at scan^2*sex	0.24	1.73	0.909	0.01	0.04
Occipital lobe left WM	Quadratic	Gestational age at scan	288.81	8.72	<0.001	0.82	0.02
		Sex	681.65	98.38	<0.001	0.28	0.03
		Gestational age at scan^2	-3.14	1.12	0.009	-0.07	0.02
		Gestational age at scan*sex	32.73	13.26	0.021	0.09	0.04
		Gestational age at scan^2*sex	-1.39	1.72	0.475	-0.03	0.04
Gyri parahippocampalis et ambiens posterior part right WM	Quadratic	Gestational age at scan	23.57	0.87	<0.001	0.82	0.03
		Sex	13.95	9.44	0.174	0.14	0.03
		Gestational age at scan^2	-0.25	0.11	0.035	-0.07	0.03
		Gestational age at scan*sex	3.22	1.29	0.019	0.11	0.04
		Gestational age at scan^2*sex	0.27	0.16	0.126	0.07	0.04

Gyri parahippocampalis et ambiens posterior part left WM	Linear	Gestational age at scan	22.94	0.55	<0.001	0.86	0.02
		Sex	11.09	6.14	0.073	0.07	0.04
		Gestational age at scan*sex	0.16	0.77	0.836	0.01	0.03
Lateral occipitotemporal gyrus fusiformis posterior part right WM	Cubic	Gestational age at scan	64.30	2.09	<0.001	1.01	0.03
		Sex	-0.15	0.03	<0.001	0.18	0.03
		Gestational age at scan^2	83.00	23.13	0.001	-0.11	0.05
		Gestational age at scan^3	-0.91	0.47	0.094	-0.27	0.06
		Gestational age at scan*sex	6.69	2.92	0.047	0.11	0.05
		Gestational age at scan^2*sex	-0.48	0.66	0.578	-0.06	0.08
		Gestational age at scan^3*sex	-0.04	0.05	0.470	-0.08	0.09
Lateral occipitotemporal gyrus fusiformis posterior part left WM	Cubic	Gestational age at scan	57.28	1.96	<0.001	0.95	0.03
		Sex	-0.12	0.03	0.001	0.18	0.03
		Gestational age at scan^2	83.87	22.23	0.001	-0.15	0.06
		Gestational age at scan^3	-1.17	0.45	0.021	-0.22	0.06
		Gestational age at scan*sex	5.44	2.75	0.089	0.09	0.05
		Gestational age at scan^2*sex	-0.76	0.63	0.320	-0.10	0.08
		Gestational age at scan^3*sex	-0.06	0.05	0.278	-0.12	0.09
Medial and inferior temporal gyri posterior part right WM	Quadratic	Gestational age at scan	118.08	4.33	<0.001	0.71	0.03
		Sex	361.93	48.22	<0.001	0.30	0.03
		Gestational age at scan^2	-3.48	0.56	<0.001	-0.16	0.03
		Gestational age at scan*sex	10.67	6.45	0.128	0.06	0.04
		Gestational age at scan^2*sex	-1.07	0.84	0.246	-0.05	0.04
Medial and inferior temporal gyri posterior part left WM	Quadratic	Gestational age at scan	90.77	4.14	<0.001	0.64	0.03
		Sex	248.37	46.85	<0.001	0.28	0.04
		Gestational age at scan^2	-4.25	0.53	<0.001	-0.23	0.03
		Gestational age at scan*sex	14.24	6.10	0.030	0.10	0.04
		Gestational age at scan^2*sex	0.18	0.79	0.846	0.01	0.04
Superior temporal gyrus posterior part right WM	Cubic	Gestational age at scan	0.24	0.04	<0.001	0.45	0.05
		Sex	91.06	26.18	0.001	0.38	0.05
		Gestational age at scan^2	1.98	0.56	0.002	0.32	0.08
		Gestational age at scan^3	0.29	3.61	0.962	0.58	0.09

		Gestational age at scan*sex	1.27	0.79	0.177	0.01	0.07
		Gestational age at scan^2*sex	0.11	0.06	0.126	0.19	0.13
		Gestational age at scan^3*sex	21.94	2.52	<0.001	0.25	0.14
Superior temporal gyrus posterior part left WM	Cubic	Gestational age at scan	0.18	0.04	<0.001	0.42	0.05
		Sex	56.37	21.41	0.018	0.29	0.04
		Gestational age at scan^2	0.58	0.47	0.310	0.09	0.08
		Gestational age at scan^3	-1.16	3.10	0.806	0.47	0.09
		Gestational age at scan*sex	1.18	0.66	0.129	-0.03	0.07
		Gestational age at scan^2*sex	0.11	0.05	0.075	0.22	0.12
		Gestational age at scan^3*sex	18.04	2.17	<0.001	0.30	0.14
Cingulate gyrus anterior part right WM	Cubic	Gestational age at scan	0.17	0.04	<0.001	0.54	0.05
		Sex	58.51	25.50	0.043	0.20	0.04
		Gestational age at scan^2	0.96	0.54	0.131	0.15	0.08
		Gestational age at scan^3	2.65	3.51	0.568	0.40	0.09
		Gestational age at scan*sex	0.43	0.77	0.688	0.05	0.07
		Gestational age at scan^2*sex	0.04	0.06	0.620	0.06	0.12
		Gestational age at scan^3*sex	27.71	2.46	<0.001	0.09	0.13
Cingulate gyrus anterior part left WM	Quadratic	Gestational age at scan	33.67	2.08	<0.001	0.75	0.03
		Sex	59.83	21.82	0.009	0.23	0.03
		Gestational age at scan^2	-1.09	0.27	<0.001	-0.15	0.03
		Gestational age at scan*sex	4.00	2.93	0.214	0.05	0.04
		Gestational age at scan^2*sex	0.07	0.38	0.879	-0.02	0.04
Cingulate gyrus posterior part right WM	Quadratic	Gestational age at scan	32.38	2.20	<0.001	0.75	0.03
		Sex	73.89	23.13	0.002	0.22	0.03
		Gestational age at scan^2	-0.89	0.29	0.004	-0.16	0.03
		Gestational age at scan*sex	-0.68	3.10	0.850	0.06	0.04
		Gestational age at scan^2*sex	-0.29	0.41	0.530	0.00	0.04
Cingulate gyrus posterior part left WM	Quadratic	Gestational age at scan	44.29	1.68	<0.001	0.78	0.02
		Sex	92.36	17.73	<0.001	0.27	0.02
		Gestational age at scan^2	-1.15	0.22	<0.001	-0.14	0.02
		Gestational age at scan*sex	3.14	2.41	0.235	0.08	0.03

		Gestational age at scan^2*sex	-0.14	0.31	0.688	-0.01	0.03
Frontal lobe right WM	Quadratic	Gestational age at scan	44.41	1.66	<0.001	1.08	0.02
		Sex	83.60	18.00	<0.001	0.13	0.02
		Gestational age at scan^2	-1.19	0.21	<0.001	0.22	0.02
		Gestational age at scan*sex	3.49	2.39	0.182	0.04	0.03
		Gestational age at scan^2*sex	-0.01	0.31	0.982	-0.02	0.03
Frontal lobe left WM	Quadratic	Gestational age at scan	997.19	27.12	<0.001	0.78	0.02
		Sex	2254.72	286.30	<0.001	0.25	0.02
		Gestational age at scan^2	-23.05	3.47	<0.001	-0.14	0.02
		Gestational age at scan*sex	106.57	37.92	0.009	0.09	0.03
		Gestational age at scan^2*sex	-2.43	4.90	0.673	-0.01	0.03
Parietal lobe right WM	Quadratic	Gestational age at scan	570.44	15.51	<0.001	0.79	0.02
		Sex	1270.93	170.94	<0.001	0.26	0.03
		Gestational age at scan^2	-12.73	2.00	<0.001	-0.14	0.02
		Gestational age at scan*sex	56.88	22.91	0.021	0.08	0.03
		Gestational age at scan^2*sex	-1.45	2.99	0.677	-0.02	0.03
Parietal lobe left WM	Quadratic	Gestational age at scan	558.17	15.59	<0.001	0.77	0.02
		Sex	1266.74	172.13	<0.001	0.27	0.02
		Gestational age at scan^2	-13.97	2.01	<0.001	-0.15	0.02
		Gestational age at scan*sex	68.88	23.00	0.005	0.10	0.03
		Gestational age at scan^2*sex	-0.21	2.99	0.953	0.00	0.03
Thalamus right WM	Quadratic	Gestational age at scan	31.12	0.65	<0.001	1.11	0.02
		Sex	14.77	6.47	0.031	0.07	0.02
		Gestational age at scan^2	0.86	0.08	<0.001	0.24	0.02
		Gestational age at scan*sex	0.17	0.91	0.874	0.01	0.03
		Gestational age at scan^2*sex	-0.08	0.12	0.562	-0.02	0.03
Thalamus left WM	Quadratic	Gestational age at scan	23.30	0.54	<0.001	1.04	0.02
		Sex	18.66	5.38	0.001	0.10	0.02
		Gestational age at scan^2	0.40	0.07	<0.001	0.14	0.02
		Gestational age at scan*sex	-0.33	0.74	0.707	-0.01	0.03
		Gestational age at scan^2*sex	-0.10	0.09	0.344	-0.03	0.03

Temporal lobe left WM (merged region)	Quadratic	Gestational age at scan	525.07	12.30	<0.001	0.84	0.02
		Sex	945.43	134.08	<0.001	0.23	0.02
		Gestational age at scan^2	-7.25	1.57	<0.001	-0.09	0.02
		Gestational age at scan*sex	42.71	17.74	0.025	0.07	0.03
		Gestational age at scan^2*sex	-0.75	2.29	0.787	-0.01	0.03
Temporal lobe right WM (merged region)	Quadratic	Gestational age at scan	555.55	12.08	<0.001	0.85	0.02
		Sex	970.26	135.93	<0.001	0.24	0.02
		Gestational age at scan^2	-5.95	1.55	<0.001	-0.07	0.02
		Gestational age at scan*sex	58.15	18.05	0.003	0.09	0.03
		Gestational age at scan^2*sex	0.11	2.33	0.967	0.00	0.03

Supplementary Table 2 - Proportional Analysis

Region	Best fitting model	Term	B	Std error	p (FDR adjusted)	Standardised B	Std Error (std beta)
Total Gray Matter	Cubic	Gestational age at scan	0.0121	0.0002	<0.001	1.1992	0.0195
		Sex	-0.0063	0.0016	<0.001	-0.0914	0.0141
		Gestational age at scan^2	-0.0001	0.0000	0.022	-0.0701	0.0280
		Gestational age at scan^3	0.0000	0.0000	<0.001	-0.3446	0.0349
		Gestational age at scan*sex	0.0004	0.0003	0.236	0.0373	0.0275
		Gestational age at scan^2*sex	0.0000	0.0001	0.949	-0.0026	0.0399
		Gestational age at scan^3*sex	0.0000	0.0000	0.655	-0.0284	0.0502
Total White Matter	Cubic	Gestational age at scan	-0.0121	0.0002	<0.001	-1.1992	0.0195
		Sex	0.0063	0.0016	<0.001	0.0914	0.0141
		Gestational age at scan^2	0.0001	0.0000	0.022	0.0701	0.0280
		Gestational age at scan^3	0.0000	0.0000	<0.001	0.3446	0.0349
		Gestational age at scan*sex	-0.0004	0.0003	0.236	-0.0373	0.0275
		Gestational age at scan^2*sex	0.0000	0.0001	0.949	0.0026	0.0399
		Gestational age at scan^3*sex	0.0000	0.0000	0.655	0.0284	0.0502
Hippocampus left	Cubic	Gestational age at scan	0.0000	0.0000	<0.001	-0.6498	0.0958
		Sex	0.0000	0.0000	0.680	-0.1704	0.0720
		Gestational age at scan^2	0.0000	0.0000	0.960	-0.0094	0.1219
		Gestational age at scan^3	0.0000	0.0000	0.050	0.4777	0.2047
		Gestational age at scan*sex	0.0000	0.0000	0.726	0.0722	0.1290
		Gestational age at scan^2*sex	0.0000	0.0000	0.626	-0.1218	0.1667
		Gestational age at scan^3*sex	0.0000	0.0000	0.911	0.0555	0.2786
Hippocampus right	Quadratic	Gestational age at scan	0.0000	0.0000	<0.001	-0.3688	0.0610
		Sex	0.0000	0.0000	0.456	-0.2320	0.0712
		Gestational age at scan^2	0.0000	0.0000	0.282	-0.1088	0.0797
		Gestational age at scan*sex	0.0000	0.0000	0.268	0.1208	0.0859
		Gestational age at scan^2*sex	0.0000	0.0000	0.180	-0.1846	0.1128
Amygdala left	Quadratic	Gestational age at scan	0.0000	0.0000	0.001	0.2113	0.0564
		Sex	0.0000	0.0000	0.539	-0.0326	0.0652
		Gestational age at scan^2	0.0000	0.0000	<0.001	-0.4006	0.0724
		Gestational age at scan*sex	0.0000	0.0000	0.429	-0.0834	0.0803

		Gestational age at scan^2*sex	0.0000	0.0000	0.423	-0.1105	0.1037
Amygdala right	Quadratic	Gestational age at scan	0.0000	0.0000	0.001	0.2075	0.0587
		Sex	0.0000	0.0000	0.996	-0.0629	0.0647
		Gestational age at scan^2	0.0000	0.0000	<0.001	-0.3445	0.0715
		Gestational age at scan*sex	0.0000	0.0000	0.806	-0.0278	0.0836
		Gestational age at scan^2*sex	0.0000	0.0000	0.575	-0.0761	0.1025
Anterior temporal lobe medial part left GM	Cubic	Gestational age at scan	0.0000	0.0000	0.082	0.2088	0.0993
		Sex	0.0000	0.0000	0.925	0.0502	0.0722
		Gestational age at scan^2	0.0000	0.0000	0.077	0.3034	0.1422
		Gestational age at scan^3	0.0000	0.0000	0.858	0.0563	0.1792
		Gestational age at scan*sex	0.0000	0.0000	0.858	-0.0426	0.1382
		Gestational age at scan^2*sex	0.0000	0.0000	0.867	0.0576	0.1999
		Gestational age at scan^3*sex	0.0000	0.0000	0.899	0.0586	0.2545
Anterior temporal lobe medial part right GM	Quadratic	Gestational age at scan	0.0000	0.0000	<0.001	0.4112	0.0713
		Sex	0.0001	0.0000	0.132	-0.0021	0.0708
		Gestational age at scan^2	0.0000	0.0000	<0.001	0.3532	0.0725
		Gestational age at scan*sex	0.0000	0.0000	0.150	-0.1846	0.1056
		Gestational age at scan^2*sex	0.0000	0.0000	0.064	-0.2353	0.1080
Anterior temporal lobe lateral part left GM	Quadratic	Gestational age at scan	0.0000	0.0000	<0.001	0.5564	0.0689
		Sex	0.0001	0.0000	0.277	0.0836	0.0674
		Gestational age at scan^2	0.0000	0.0000	<0.001	0.2758	0.0687
		Gestational age at scan*sex	0.0000	0.0000	0.852	-0.0237	0.0964
		Gestational age at scan^2*sex	0.0000	0.0000	0.559	-0.0756	0.0967
Anterior temporal lobe lateral part right GM	Quadratic	Gestational age at scan	0.0001	0.0000	<0.001	0.8734	0.0632
		Sex	0.0000	0.0000	0.334	-0.0413	0.0577
		Gestational age at scan^2	0.0000	0.0000	<0.001	0.4116	0.0631
		Gestational age at scan*sex	0.0000	0.0000	0.168	-0.1492	0.0890
		Gestational age at scan^2*sex	0.0000	0.0000	0.069	-0.1912	0.0891
Gyri parahippocampalis et ambiens anterior part left GM	Cubic	Gestational age at scan	0.0000	0.0000	0.002	0.3570	0.1025
		Sex	0.0000	0.0001	0.633	-0.1243	0.0710
		Gestational age at scan^2	0.0000	0.0000	0.005	-0.4687	0.1474

		Gestational age at scan^3	0.0000	0.0000	<0.001	-0.7753	0.1842
		Gestational age at scan*sex	0.0000	0.0000	0.415	-0.1542	0.1359
		Gestational age at scan^2*sex	0.0000	0.0000	0.314	-0.2671	0.1978
		Gestational age at scan^3*sex	0.0000	0.0000	0.952	-0.0252	0.2493
Gyri parahippocampalis et ambiens anterior part right GM	Cubic	Gestational age at scan	0.0000	0.0000	<0.001	0.6745	0.0998
		Sex	0.0000	0.0000	0.427	-0.2617	0.0687
		Gestational age at scan^2	0.0000	0.0000	0.013	-0.3863	0.1348
		Gestational age at scan^3	0.0000	0.0000	<0.001	-0.9799	0.1819
		Gestational age at scan*sex	0.0000	0.0000	0.897	-0.0319	0.1334
		Gestational age at scan^2*sex	0.0000	0.0000	0.568	-0.1565	0.1827
		Gestational age at scan^3*sex	0.0000	0.0000	0.904	0.0530	0.2478
Superior temporal gyrus middle part left GM	Cubic	Gestational age at scan	0.0002	0.0000	<0.001	0.8682	0.0384
		Sex	0.0001	0.0001	0.601	-0.0140	0.0290
		Gestational age at scan^2	0.0000	0.0000	0.026	-0.1532	0.0587
		Gestational age at scan^3	0.0000	0.0000	0.162	-0.1224	0.0701
		Gestational age at scan*sex	0.0000	0.0000	0.143	0.0957	0.0529
		Gestational age at scan^2*sex	0.0000	0.0000	0.193	-0.1340	0.0814
		Gestational age at scan^3*sex	0.0000	0.0000	0.170	-0.1692	0.0984
Superior temporal gyrus middle part right GM	Cubic	Gestational age at scan	0.0002	0.0000	<0.001	0.8991	0.0386
		Sex	0.0001	0.0001	0.321	-0.0226	0.0298
		Gestational age at scan^2	0.0000	0.0000	0.061	-0.1356	0.0604
		Gestational age at scan^3	0.0000	0.0000	0.120	-0.1345	0.0706
		Gestational age at scan*sex	0.0000	0.0000	0.125	0.1008	0.0536
		Gestational age at scan^2*sex	0.0000	0.0000	0.017	-0.2346	0.0844
		Gestational age at scan^3*sex	0.0000	0.0000	0.010	-0.2975	0.1001
Medial and inferior temporal gyri anterior part left GM	Cubic	Gestational age at scan	0.0002	0.0000	<0.001	1.2482	0.0606
		Sex	0.0000	0.0001	0.842	0.0381	0.0483
		Gestational age at scan^2	0.0000	0.0000	0.002	0.3384	0.0983
		Gestational age at scan^3	0.0000	0.0000	<0.001	-0.4679	0.1130
		Gestational age at scan*sex	0.0000	0.0000	0.377	0.0995	0.0823
		Gestational age at scan^2*sex	0.0000	0.0000	0.741	0.0709	0.1337

		Gestational age at scan^3*sex	0.0000	0.0000	0.901	-0.0347	0.1563
Medial and inferior temporal gyri anterior part right GM	Cubic	Gestational age at scan	0.0002	0.0000	<0.001	1.2540	0.0536
		Sex	-0.0001	0.0001	0.363	-0.0462	0.0428
		Gestational age at scan^2	0.0000	0.0000	<0.001	0.3867	0.0846
		Gestational age at scan^3	0.0000	0.0000	0.005	-0.3237	0.1000
		Gestational age at scan*sex	0.0000	0.0000	0.189	0.1214	0.0732
		Gestational age at scan^2*sex	0.0000	0.0000	0.881	0.0309	0.1157
		Gestational age at scan^3*sex	0.0000	0.0000	0.769	-0.0656	0.1391
Lateral occipitotemporal gyrus fusiformis anterior part left GM	Quadratic	Gestational age at scan	0.0000	0.0000	<0.001	0.3959	0.0660
		Sex	0.0000	0.0000	0.932	-0.0151	0.0581
		Gestational age at scan^2	0.0000	0.0000	<0.001	-0.2610	0.0659
		Gestational age at scan*sex	0.0000	0.0000	0.567	-0.0677	0.0884
		Gestational age at scan^2*sex	0.0000	0.0000	0.798	-0.0311	0.0887
Lateral occipitotemporal gyrus fusiformis anterior part right GM	Linear	Gestational age at scan	0.0000	0.0000	<0.001	0.6143	0.0382
		Sex	0.0000	0.0000	0.313	-0.0642	0.0539
		Gestational age at scan*sex	0.0000	0.0000	0.137	0.0904	0.0530
Cerebellum left	Linear	Gestational age at scan	0.0011	0.0000	<0.001	0.9497	0.0151
		Sex	-0.0009	0.0002	<0.001	-0.1190	0.0235
		Gestational age at scan*sex	0.0000	0.0000	0.493	-0.0176	0.0207
Cerebellum right	Linear	Gestational age at scan	0.0010	0.0000	<0.001	0.9260	0.0178
		Sex	-0.0009	0.0002	<0.001	-0.1286	0.0273
		Gestational age at scan*sex	0.0000	0.0000	0.723	0.0111	0.0239
Brainstem	Cubic	Gestational age at scan	-0.0005	0.0000	<0.001	-0.6343	0.0259
		Sex	-0.0007	0.0001	<0.001	-0.1161	0.0196
		Gestational age at scan^2	0.0000	0.0000	<0.001	0.2388	0.0344
		Gestational age at scan^3	0.0000	0.0000	0.035	-0.1296	0.0520
		Gestational age at scan*sex	0.0000	0.0000	0.769	0.0172	0.0363
		Gestational age at scan^2*sex	0.0000	0.0000	0.627	0.0354	0.0487
		Gestational age at scan^3*sex	0.0000	0.0000	0.995	0.0005	0.0740
Insula right GM	Cubic	Gestational age at scan	0.0000	0.0000	<0.001	0.3776	0.0473
		Sex	0.0001	0.0000	0.393	0.0018	0.0401

		Gestational age at scan^2	0.0000	0.0000	0.539	-0.0707	0.0775
		Gestational age at scan^3	0.0000	0.0000	<0.001	0.4009	0.0900
		Gestational age at scan*sex	0.0000	0.0000	0.958	-0.0055	0.0650
		Gestational age at scan^2*sex	0.0000	0.0000	0.482	-0.1074	0.1060
		Gestational age at scan^3*sex	0.0000	0.0000	0.904	-0.0270	0.1257
Insula left GM	Quadratic	Gestational age at scan	0.0001	0.0000	<0.001	0.4993	0.0396
		Sex	-0.0001	0.0000	0.402	-0.0100	0.0411
		Gestational age at scan^2	0.0000	0.0000	<0.001	-0.3702	0.0396
		Gestational age at scan*sex	0.0000	0.0000	0.089	-0.0128	0.0533
		Gestational age at scan^2*sex	0.0000	0.0000	0.515	-0.0773	0.0538
Occipital lobe right GM	Linear	Gestational age at scan	0.0007	0.0000	<0.001	0.8521	0.0256
		Sex	-0.0005	0.0002	0.005	-0.1075	0.0356
		Gestational age at scan*sex	0.0000	0.0000	0.267	0.0454	0.0349
Occipital lobe left GM	Cubic	Gestational age at scan	0.0009	0.0000	<0.001	1.0519	0.0403
		Sex	-0.0002	0.0003	0.741	-0.0829	0.0318
		Gestational age at scan^2	0.0000	0.0000	0.942	-0.0076	0.0628
		Gestational age at scan^3	0.0000	0.0000	0.062	-0.1680	0.0750
		Gestational age at scan*sex	0.0000	0.0000	0.984	0.0020	0.0549
		Gestational age at scan^2*sex	0.0000	0.0000	0.323	-0.1137	0.0857
		Gestational age at scan^3*sex	0.0000	0.0000	0.385	-0.1236	0.1040
Gyri parahippocampalis et ambiens posterior part right GM	Cubic	Gestational age at scan	0.0001	0.0000	<0.001	1.0667	0.0737
		Sex	0.0000	0.0000	0.490	-0.1400	0.0524
		Gestational age at scan^2	0.0000	0.0000	0.001	-0.4118	0.1060
		Gestational age at scan^3	0.0000	0.0000	<0.001	-0.8276	0.1315
		Gestational age at scan*sex	0.0000	0.0000	0.118	-0.1962	0.1024
		Gestational age at scan^2*sex	0.0000	0.0000	0.942	0.0180	0.1494
		Gestational age at scan^3*sex	0.0000	0.0000	0.477	0.1907	0.1867
Gyri parahippocampalis et ambiens posterior part left GM	Cubic	Gestational age at scan	0.0000	0.0000	<0.001	0.7402	0.0797
		Sex	-0.0001	0.0000	0.186	-0.2455	0.0567
		Gestational age at scan^2	0.0000	0.0000	0.003	-0.4021	0.1174
		Gestational age at scan^3	0.0000	0.0000	0.002	-0.5065	0.1440

		Gestational age at scan*sex	0.0000	0.0000	0.798	-0.0443	0.1064
		Gestational age at scan^2*sex	0.0000	0.0000	0.536	-0.1459	0.1584
		Gestational age at scan^3*sex	0.0000	0.0000	0.675	-0.1250	0.1962
Lateral occipitotemporal gyrus fusiformis posterior part right GM	Cubic	Gestational age at scan	0.0001	0.0000	<0.001	1.2307	0.0411
		Sex	0.0000	0.0000	0.682	-0.0490	0.0312
		Gestational age at scan^2	0.0000	0.0000	0.001	-0.2400	0.0657
		Gestational age at scan^3	0.0000	0.0000	<0.001	-0.6089	0.0752
		Gestational age at scan*sex	0.0000	0.0000	0.536	-0.0510	0.0554
		Gestational age at scan^2*sex	0.0000	0.0000	0.956	-0.0078	0.0893
		Gestational age at scan^3*sex	0.0000	0.0000	0.939	0.0153	0.1034
Lateral occipitotemporal gyrus fusiformis posterior part left GM	Cubic	Gestational age at scan	0.0001	0.0000	<0.001	1.0696	0.0480
		Sex	0.0000	0.0001	0.940	-0.0324	0.0363
		Gestational age at scan^2	0.0000	0.0000	0.001	-0.2861	0.0767
		Gestational age at scan^3	0.0000	0.0000	<0.001	-0.4930	0.0875
		Gestational age at scan*sex	0.0000	0.0000	0.934	0.0104	0.0647
		Gestational age at scan^2*sex	0.0000	0.0000	0.606	-0.0810	0.1044
		Gestational age at scan^3*sex	0.0000	0.0000	0.420	-0.1355	0.1205
Medial and inferior temporal gyri posterior part right GM	Cubic	Gestational age at scan	0.0003	0.0000	<0.001	1.3511	0.0466
		Sex	0.0003	0.0001	0.002	0.1338	0.0364
		Gestational age at scan^2	0.0000	0.0000	0.029	0.1803	0.0704
		Gestational age at scan^3	0.0000	0.0000	<0.001	-0.4892	0.0861
		Gestational age at scan*sex	0.0000	0.0000	0.939	-0.0095	0.0649
		Gestational age at scan^2*sex	0.0000	0.0000	0.299	-0.1359	0.0983
		Gestational age at scan^3*sex	0.0000	0.0000	0.578	-0.1018	0.1221
Medial and inferior temporal gyri posterior part left GM	Cubic	Gestational age at scan	0.0004	0.0000	<0.001	1.4192	0.0493
		Sex	0.0001	0.0001	0.626	0.0875	0.0363
		Gestational age at scan^2	0.0000	0.0000	0.300	0.1025	0.0743
		Gestational age at scan^3	0.0000	0.0000	<0.001	-0.6965	0.0893
		Gestational age at scan*sex	0.0000	0.0000	0.955	0.0061	0.0677
		Gestational age at scan^2*sex	0.0000	0.0000	0.576	0.0864	0.1029
		Gestational age at scan^3*sex	0.0000	0.0000	0.601	0.0989	0.1250

Superior temporal gyrus posterior part right GM	Linear	Gestational age at scan	0.0001	0.0000	<0.001	0.8670	0.0225
		Sex	0.0000	0.0000	0.443	-0.0329	0.0350
		Gestational age at scan*sex	0.0000	0.0000	0.846	0.0081	0.0311
Superior temporal gyrus posterior part left GM	Quadratic	Gestational age at scan	0.0001	0.0000	<0.001	0.7346	0.0351
		Sex	-0.0001	0.0000	0.022	-0.0736	0.0356
		Gestational age at scan^2	0.0000	0.0000	<0.001	-0.1580	0.0354
		Gestational age at scan*sex	0.0000	0.0000	0.123	0.0920	0.0495
		Gestational age at scan^2*sex	0.0000	0.0000	0.205	0.0790	0.0504
Cingulate gyrus anterior part right GM	Linear	Gestational age at scan	0.0000	0.0000	<0.001	0.5017	0.0396
		Sex	0.0000	0.0000	0.501	0.0514	0.0620
		Gestational age at scan*sex	0.0000	0.0000	0.251	0.0766	0.0568
Cingulate gyrus anterior part left GM	Linear	Gestational age at scan	0.0000	0.0000	<0.001	0.3379	0.0453
		Sex	0.0000	0.0000	0.550	-0.0510	0.0681
		Gestational age at scan*sex	0.0000	0.0000	0.832	0.0180	0.0643
Cingulate gyrus posterior part right GM	Quadratic	Gestational age at scan	0.0001	0.0000	<0.001	0.7117	0.0517
		Sex	0.0000	0.0001	0.567	0.0898	0.0494
		Gestational age at scan^2	0.0000	0.0000	0.954	0.0035	0.0516
		Gestational age at scan*sex	0.0000	0.0000	0.053	0.1657	0.0731
		Gestational age at scan^2*sex	0.0000	0.0000	0.031	0.1828	0.0732
Cingulate gyrus posterior part left GM	Linear	Gestational age at scan	0.0001	0.0000	<0.001	0.7615	0.0316
		Sex	0.0000	0.0000	0.546	0.0351	0.0462
		Gestational age at scan*sex	0.0000	0.0000	0.688	0.0227	0.0429
Frontal lobe right GM	Linear	Gestational age at scan	0.0012	0.0000	<0.001	0.9010	0.0182
		Sex	0.0001	0.0002	0.632	0.0163	0.0261
		Gestational age at scan*sex	0.0001	0.0000	0.039	0.0585	0.0253
Frontal lobe left GM	Quadratic	Gestational age at scan	0.0012	0.0000	<0.001	0.9523	0.0292
		Sex	-0.0002	0.0004	0.595	-0.0194	0.0268
		Gestational age at scan^2	0.0000	0.0000	0.027	0.0745	0.0292
		Gestational age at scan*sex	0.0001	0.0001	0.152	0.0704	0.0405
		Gestational age at scan^2*sex	0.0000	0.0000	0.815	0.0128	0.0407
Parietal lobe right GM	Cubic	Gestational age at scan	0.0014	0.0000	<0.001	1.1385	0.0266

		Sex	-0.0011	0.0003	0.001	-0.1075	0.0215
		Gestational age at scan^2	0.0000	0.0000	0.630	-0.0304	0.0423
		Gestational age at scan^3	0.0000	0.0000	<0.001	-0.2378	0.0495
		Gestational age at scan*sex	0.0000	0.0000	0.734	0.0203	0.0372
		Gestational age at scan^2*sex	0.0000	0.0000	0.771	0.0276	0.0592
		Gestational age at scan^3*sex	0.0000	0.0000	0.826	-0.0264	0.0704
Parietal lobe left GM	Cubic	Gestational age at scan	0.0014	0.0000	<0.001	1.1394	0.0272
		Sex	-0.0011	0.0003	<0.001	-0.0984	0.0208
		Gestational age at scan^2	0.0000	0.0000	0.626	-0.0307	0.0416
		Gestational age at scan^3	0.0000	0.0000	<0.001	-0.2557	0.0497
		Gestational age at scan*sex	0.0000	0.0000	0.750	0.0193	0.0378
		Gestational age at scan^2*sex	0.0000	0.0000	0.264	0.0849	0.0582
		Gestational age at scan^3*sex	0.0000	0.0000	0.518	0.0671	0.0705
Temporal lobe right GM (merged region)	Cubic	Gestational age at scan	0.0013	0.0000	<0.001	1.2842	0.0322
		Sex	0.0003	0.0003	0.400	-0.0229	0.0242
		Gestational age at scan^2	0.0000	0.0000	0.674	0.0310	0.0484
		Gestational age at scan^3	0.0000	0.0000	<0.001	-0.4163	0.0594
		Gestational age at scan*sex	0.0000	0.0000	0.920	0.0080	0.0430
		Gestational age at scan^2*sex	0.0000	0.0000	0.127	-0.1218	0.0651
		Gestational age at scan^3*sex	0.0000	0.0000	0.412	-0.0921	0.0808
Temporal lobe left GM (merged region)	Cubic	Gestational age at scan	0.0008	0.0000	<0.001	1.1108	0.0392
		Sex	0.0000	0.0002	0.939	-0.0370	0.0292
		Gestational age at scan^2	0.0000	0.0000	0.117	-0.1132	0.0588
		Gestational age at scan^3	0.0000	0.0000	<0.001	-0.3563	0.0717
		Gestational age at scan*sex	0.0000	0.0000	0.633	0.0376	0.0529
		Gestational age at scan^2*sex	0.0000	0.0000	0.559	-0.0699	0.0799
		Gestational age at scan^3*sex	0.0000	0.0000	0.560	-0.0862	0.0987
Caudate nucleus right	Cubic	Gestational age at scan	0.0001	0.0000	<0.001	0.9582	0.0739
		Sex	0.0000	0.0001	0.897	0.0209	0.0547
		Gestational age at scan^2	0.0000	0.0000	<0.001	-0.6906	0.1114
		Gestational age at scan^3	0.0000	0.0000	<0.001	-0.9255	0.1330

		Gestational age at scan*sex	0.0000	0.0000	0.069	-0.2247	0.1028
		Gestational age at scan^2*sex	0.0000	0.0000	0.993	-0.0020	0.1569
		Gestational age at scan^3*sex	0.0000	0.0000	0.985	0.0063	0.1892
Caudate nucleus left	Cubic	Gestational age at scan	0.0001	0.0000	<0.001	1.0117	0.0715
		Sex	0.0000	0.0001	0.858	-0.0078	0.0553
		Gestational age at scan^2	0.0000	0.0000	<0.001	-0.6599	0.1100
		Gestational age at scan^3	0.0000	0.0000	<0.001	-0.9795	0.1302
		Gestational age at scan*sex	0.0000	0.0000	0.041	-0.2442	0.1011
		Gestational age at scan^2*sex	0.0000	0.0000	0.752	0.0794	0.1569
		Gestational age at scan^3*sex	0.0000	0.0000	0.657	0.1261	0.1881
Thalamus right	Cubic	Gestational age at scan	0.0003	0.0000	<0.001	1.3912	0.0594
		Sex	-0.0004	0.0001	<0.001	-0.2148	0.0466
		Gestational age at scan^2	0.0000	0.0000	<0.001	-1.1163	0.0805
		Gestational age at scan^3	0.0000	0.0000	<0.001	-2.1437	0.1202
		Gestational age at scan*sex	0.0000	0.0000	0.984	-0.0032	0.0846
		Gestational age at scan^2*sex	0.0000	0.0000	0.582	0.0948	0.1152
		Gestational age at scan^3*sex	0.0000	0.0000	0.827	-0.0646	0.1736
Thalamus left	Cubic	Gestational age at scan	0.0003	0.0000	<0.001	1.3032	0.0593
		Sex	-0.0003	0.0001	<0.001	-0.1408	0.0471
		Gestational age at scan^2	0.0000	0.0000	<0.001	-0.9720	0.0791
		Gestational age at scan^3	0.0000	0.0000	<0.001	-2.0350	0.1278
		Gestational age at scan*sex	0.0000	0.0000	0.767	-0.0395	0.0821
		Gestational age at scan^2*sex	0.0000	0.0000	0.567	0.0950	0.1104
		Gestational age at scan^3*sex	0.0000	0.0000	0.654	-0.1214	0.1787
Subthalamic nucleus right	Cubic	Gestational age at scan	0.0000	0.0000	<0.001	-1.0228	0.0506
		Sex	0.0000	0.0000	0.082	-0.0231	0.0448
		Gestational age at scan^2	0.0000	0.0000	<0.001	0.5414	0.0611
		Gestational age at scan^3	0.0000	0.0000	<0.001	0.7699	0.1166
		Gestational age at scan*sex	0.0000	0.0000	0.031	0.1814	0.0714
		Gestational age at scan^2*sex	0.0000	0.0000	0.901	-0.0193	0.0866
		Gestational age at scan^3*sex	0.0000	0.0000	0.361	-0.2082	0.1672

Subthalamic nucleus left	Cubic	Gestational age at scan	0.0000	0.0000	<0.001	-0.9193	0.0400
		Sex	0.0000	0.0000	0.178	-0.0714	0.0406
		Gestational age at scan^2	0.0000	0.0000	<0.001	0.3933	0.0514
		Gestational age at scan^3	0.0000	0.0000	<0.001	0.4252	0.1032
		Gestational age at scan*sex	0.0000	0.0000	0.109	0.1099	0.0560
		Gestational age at scan^2*sex	0.0000	0.0000	0.576	-0.0596	0.0708
		Gestational age at scan^3*sex	0.0000	0.0000	0.663	-0.0948	0.1439
Lentiform nucleus right	Cubic	Gestational age at scan	0.0000	0.0000	0.110	0.1565	0.0801
		Sex	-0.0001	0.0001	0.619	-0.1948	0.0559
		Gestational age at scan^2	0.0000	0.0000	0.661	-0.0762	0.1147
		Gestational age at scan^3	0.0000	0.0000	<0.001	-0.8692	0.1434
		Gestational age at scan*sex	0.0000	0.0000	0.303	0.1486	0.1083
		Gestational age at scan^2*sex	0.0000	0.0000	0.210	-0.2508	0.1572
		Gestational age at scan^3*sex	0.0000	0.0000	0.328	-0.2610	0.1980
Lentiform nucleus left	Cubic	Gestational age at scan	0.0000	0.0000	0.277	0.1236	0.0864
		Sex	-0.0002	0.0001	0.347	-0.1897	0.0626
		Gestational age at scan^2	0.0000	0.0000	0.004	-0.4104	0.1249
		Gestational age at scan^3	0.0000	0.0000	<0.001	-1.0023	0.1569
		Gestational age at scan*sex	0.0000	0.0000	0.380	0.1411	0.1173
		Gestational age at scan^2*sex	0.0000	0.0000	0.528	-0.1596	0.1711
		Gestational age at scan^3*sex	0.0000	0.0000	0.445	-0.2328	0.2172
Corpus Callosum	Cubic	Gestational age at scan	0.0003	0.0000	<0.001	1.0632	0.0689
		Sex	-0.0002	0.0001	0.193	-0.0123	0.0491
		Gestational age at scan^2	0.0000	0.0000	0.134	-0.1820	0.0986
		Gestational age at scan^3	0.0000	0.0000	<0.001	-1.5820	0.1234
		Gestational age at scan*sex	-0.0001	0.0000	0.051	-0.2218	0.0953
		Gestational age at scan^2*sex	0.0000	0.0000	0.065	0.3064	0.1382
		Gestational age at scan^3*sex	0.0000	0.0000	0.067	0.3840	0.1744
Lateral Ventricle left	Quadratic	Gestational age at scan	-0.0007	0.0000	<0.001	-0.5933	0.0361
		Sex	-0.0007	0.0004	0.087	0.0960	0.0470
		Gestational age at scan^2	0.0000	0.0000	<0.001	0.2025	0.0514

		Gestational age at scan*sex	0.0000	0.0001	0.889	0.0092	0.0525
		Gestational age at scan^2*sex	0.0000	0.0000	0.005	0.2373	0.0749
Lateral Ventricle right	Quadratic	Gestational age at scan	-0.0005	0.0000	<0.001	-0.5784	0.0300
		Sex	-0.0002	0.0003	0.658	0.1135	0.0434
		Gestational age at scan^2	0.0000	0.0000	<0.001	0.2127	0.0448
		Gestational age at scan*sex	0.0000	0.0000	0.417	-0.0499	0.0463
		Gestational age at scan^2*sex	0.0000	0.0000	0.034	0.1698	0.0692
Anterior temporal lobe medial part left WM	Linear	Gestational age at scan	0.0000	0.0000	<0.001	0.4238	0.0445
		Sex	0.0000	0.0000	0.912	0.0103	0.0658
		Gestational age at scan*sex	0.0000	0.0000	0.659	-0.0357	0.0607
Anterior temporal lobe medial part right WM	Linear	Gestational age at scan	0.0000	0.0000	<0.001	0.2152	0.0440
		Sex	0.0000	0.0000	0.720	0.0329	0.0694
		Gestational age at scan*sex	0.0000	0.0000	0.864	-0.0140	0.0625
Anterior temporal lobe lateral part left WM	Linear	Gestational age at scan	0.0000	0.0000	<0.001	0.7431	0.0297
		Sex	0.0000	0.0000	0.310	0.0609	0.0507
		Gestational age at scan*sex	0.0000	0.0000	0.400	-0.0431	0.0424
Anterior temporal lobe lateral part right WM	Linear	Gestational age at scan	0.0000	0.0000	<0.001	0.7249	0.0317
		Sex	0.0000	0.0000	0.081	-0.1061	0.0542
		Gestational age at scan*sex	0.0000	0.0000	0.082	-0.0870	0.0441
Gyri parahippocampalis et ambiens anterior part left WM	Cubic	Gestational age at scan	0.0000	0.0000	<0.001	0.6786	0.0947
		Sex	0.0000	0.0000	0.237	-0.0329	0.0743
		Gestational age at scan^2	0.0000	0.0000	0.010	-0.3802	0.1279
		Gestational age at scan^3	0.0000	0.0000	<0.001	-0.8602	0.1886
		Gestational age at scan*sex	0.0000	0.0000	0.329	0.1808	0.1378
		Gestational age at scan^2*sex	0.0000	0.0000	0.882	0.0483	0.1859
		Gestational age at scan^3*sex	0.0000	0.0000	0.559	-0.2435	0.2779
Gyri parahippocampalis et ambiens anterior part right WM	Cubic	Gestational age at scan	0.0000	0.0000	<0.001	0.6325	0.0802
		Sex	0.0000	0.0000	0.382	-0.0222	0.0671
		Gestational age at scan^2	0.0000	0.0000	<0.001	-0.5927	0.1116
		Gestational age at scan^3	0.0000	0.0000	<0.001	-0.8059	0.1490
		Gestational age at scan*sex	0.0000	0.0000	0.858	0.0402	0.1307

		Gestational age at scan^2*sex	0.0000	0.0000	0.655	0.1207	0.1784
		Gestational age at scan^3*sex	0.0000	0.0000	0.989	0.0064	0.2455
Superior temporal gyrus middle part left WM	Cubic	Gestational age at scan	-0.0003	0.0000	<0.001	-1.2154	0.0587
		Sex	0.0002	0.0001	0.201	0.0002	0.0442
		Gestational age at scan^2	0.0000	0.0000	0.008	0.2637	0.0866
		Gestational age at scan^3	0.0000	0.0000	<0.001	0.7762	0.1067
		Gestational age at scan*sex	0.0000	0.0000	0.682	0.0514	0.0824
		Gestational age at scan^2*sex	0.0000	0.0000	0.065	-0.2714	0.1224
		Gestational age at scan^3*sex	0.0000	0.0000	0.095	-0.3093	0.1528
Superior temporal gyrus middle part right WM	Cubic	Gestational age at scan	-0.0002	0.0000	<0.001	-0.9374	0.0488
		Sex	-0.0001	0.0001	0.630	-0.0555	0.0363
		Gestational age at scan^2	0.0000	0.0000	0.003	0.2261	0.0659
		Gestational age at scan^3	0.0000	0.0000	0.011	0.2768	0.0943
		Gestational age at scan*sex	0.0001	0.0000	0.013	0.1940	0.0676
		Gestational age at scan^2*sex	0.0000	0.0000	0.120	-0.1756	0.0922
		Gestational age at scan^3*sex	0.0000	0.0000	0.011	-0.3879	0.1328
Medial and inferior temporal gyri anterior part left WM	Quadratic	Gestational age at scan	-0.0002	0.0000	<0.001	-0.8138	0.0434
		Sex	0.0001	0.0001	0.358	-0.0023	0.0474
		Gestational age at scan^2	0.0000	0.0000	0.125	-0.0878	0.0476
		Gestational age at scan*sex	0.0000	0.0000	0.092	-0.1262	0.0630
		Gestational age at scan^2*sex	0.0000	0.0000	0.274	-0.0963	0.0696
Medial and inferior temporal gyri anterior part right WM	Linear	Gestational age at scan	-0.0002	0.0000	<0.001	-0.7638	0.0336
		Sex	-0.0001	0.0001	0.145	0.0247	0.0464
		Gestational age at scan*sex	0.0000	0.0000	0.659	-0.0754	0.0491
Lateral occipitotemporal gyrus fusiformis anterior part left WM	Cubic	Gestational age at scan	0.0001	0.0000	<0.001	0.7379	0.0873
		Sex	0.0000	0.0000	0.911	0.1096	0.0713
		Gestational age at scan^2	0.0000	0.0000	0.047	-0.2835	0.1201
		Gestational age at scan^3	0.0000	0.0000	<0.001	-1.0946	0.1817
		Gestational age at scan*sex	0.0000	0.0000	0.625	0.0931	0.1257
		Gestational age at scan^2*sex	0.0000	0.0000	0.836	0.0623	0.1728
		Gestational age at scan^3*sex	0.0000	0.0000	0.771	-0.1225	0.2641

Lateral occipitotemporal gyrus fusiformis anterior part right WM	Cubic	Gestational age at scan	0.0000	0.0000	0.925	0.0142	0.0813
		Sex	0.0000	0.0000	0.858	0.1234	0.0696
		Gestational age at scan^2	0.0000	0.0000	0.767	-0.0551	0.1147
		Gestational age at scan^3	0.0000	0.0000	0.009	-0.5216	0.1744
		Gestational age at scan*sex	0.0000	0.0000	0.602	0.0922	0.1174
		Gestational age at scan^2*sex	0.0000	0.0000	0.626	0.1213	0.1650
		Gestational age at scan^3*sex	0.0000	0.0000	0.966	0.0165	0.2535
Insula right WM	Quadratic	Gestational age at scan	-0.0001	0.0000	<0.001	-0.8393	0.0623
		Sex	0.0000	0.0001	0.717	-0.0163	0.0647
		Gestational age at scan^2	0.0000	0.0000	<0.001	-0.5854	0.0674
		Gestational age at scan*sex	0.0000	0.0000	0.269	0.1246	0.0889
		Gestational age at scan^2*sex	0.0000	0.0000	0.798	0.0344	0.0969
Insula left WM	Quadratic	Gestational age at scan	-0.0001	0.0000	<0.001	-0.8152	0.0670
		Sex	0.0000	0.0001	0.801	-0.0630	0.0620
		Gestational age at scan^2	0.0000	0.0000	<0.001	-0.6207	0.0683
		Gestational age at scan*sex	0.0000	0.0000	0.505	0.0837	0.0940
		Gestational age at scan^2*sex	0.0000	0.0000	0.772	-0.0387	0.0963
Occipital lobe right WM	Cubic	Gestational age at scan	-0.0007	0.0000	<0.001	-1.4159	0.0623
		Sex	0.0005	0.0003	0.140	0.1512	0.0478
		Gestational age at scan^2	0.0000	0.0000	0.618	0.0707	0.0941
		Gestational age at scan^3	0.0000	0.0000	<0.001	0.9642	0.1148
		Gestational age at scan*sex	0.0001	0.0000	0.078	0.1815	0.0855
		Gestational age at scan^2*sex	0.0000	0.0000	0.766	-0.0631	0.1296
		Gestational age at scan^3*sex	0.0000	0.0000	0.447	-0.1713	0.1605
Occipital lobe left WM	Cubic	Gestational age at scan	-0.0005	0.0000	<0.001	-1.1347	0.0693
		Sex	0.0009	0.0003	0.001	0.2270	0.0565
		Gestational age at scan^2	0.0000	0.0000	0.728	0.0577	0.1039
		Gestational age at scan^3	0.0000	0.0000	<0.001	0.8155	0.1277
		Gestational age at scan*sex	0.0000	0.0000	0.771	0.0480	0.1035
		Gestational age at scan^2*sex	0.0000	0.0000	0.328	-0.2037	0.1549
		Gestational age at scan^3*sex	0.0000	0.0000	0.540	-0.1760	0.1936

Gyri parahippocampalis et ambiens posterior part right WM	Linear	Gestational age at scan	0.0000	0.0000	<0.001	-0.5451	0.0404
		Sex	0.0000	0.0000	0.116	-0.1029	0.0578
		Gestational age at scan*sex	0.0000	0.0000	0.203	-0.0842	0.0569
Gyri parahippocampalis et ambiens posterior part left WM	Linear	Gestational age at scan	0.0000	0.0000	<0.001	-0.5678	0.0385
		Sex	-0.0001	0.0000	0.001	-0.2010	0.0574
		Gestational age at scan*sex	0.0000	0.0000	0.208	-0.0781	0.0534
Lateral occipitotemporal gyrus fusiformis posterior part right WM	Cubic	Gestational age at scan	0.0001	0.0000	<0.001	0.9074	0.0768
		Sex	0.0001	0.0001	0.343	0.0571	0.0590
		Gestational age at scan^2	0.0000	0.0000	<0.001	-0.7230	0.1192
		Gestational age at scan^3	0.0000	0.0000	<0.001	-1.1333	0.1409
		Gestational age at scan*sex	0.0000	0.0000	0.867	-0.0305	0.1057
		Gestational age at scan^2*sex	0.0000	0.0000	0.576	-0.1390	0.1650
		Gestational age at scan^3*sex	0.0000	0.0000	0.744	-0.1036	0.1978
Lateral occipitotemporal gyrus fusiformis posterior part left WM	Cubic	Gestational age at scan	0.0000	0.0000	<0.001	0.4044	0.0882
		Sex	0.0001	0.0001	0.520	0.0491	0.0667
		Gestational age at scan^2	0.0000	0.0000	<0.001	-0.8677	0.1349
		Gestational age at scan^3	0.0000	0.0000	<0.001	-0.8824	0.1618
		Gestational age at scan*sex	0.0000	0.0000	0.952	-0.0119	0.1200
		Gestational age at scan^2*sex	0.0000	0.0000	0.576	-0.1549	0.1845
		Gestational age at scan^3*sex	0.0000	0.0000	0.554	-0.1984	0.2243
Medial and inferior temporal gyri posterior part right WM	Linear	Gestational age at scan	-0.0003	0.0000	<0.001	-0.8670	0.0282
		Sex	0.0004	0.0001	<0.001	0.1767	0.0382
		Gestational age at scan*sex	0.0000	0.0000	0.193	-0.0595	0.0395
Medial and inferior temporal gyri posterior part left WM	Cubic	Gestational age at scan	-0.0004	0.0000	<0.001	-0.8893	0.0373
		Sex	0.0000	0.0001	0.952	0.0612	0.0285
		Gestational age at scan^2	0.0000	0.0000	0.515	0.0515	0.0536
		Gestational age at scan^3	0.0000	0.0000	0.788	0.0301	0.0687
		Gestational age at scan*sex	-0.0001	0.0000	0.035	-0.1317	0.0529
		Gestational age at scan^2*sex	0.0000	0.0000	0.036	0.1876	0.0760
		Gestational age at scan^3*sex	0.0000	0.0000	0.018	0.2729	0.0990
Superior temporal gyrus posterior part right WM	Cubic	Gestational age at scan	-0.0002	0.0000	<0.001	-0.4851	0.0342

		Sex	0.0001	0.0001	0.321	0.0727	0.0274
		Gestational age at scan^2	0.0000	0.0000	<0.001	0.3827	0.0444
		Gestational age at scan^3	0.0000	0.0000	0.125	-0.1377	0.0731
		Gestational age at scan*sex	0.0000	0.0000	0.010	-0.1455	0.0491
		Gestational age at scan^2*sex	0.0000	0.0000	0.090	0.1311	0.0638
		Gestational age at scan^3*sex	0.0000	0.0000	0.059	0.2398	0.1061
Superior temporal gyrus posterior part left WM	Cubic	Gestational age at scan	-0.0002	0.0000	<0.001	-0.8593	0.0422
		Sex	0.0000	0.0001	0.770	0.0227	0.0316
		Gestational age at scan^2	0.0000	0.0000	<0.001	0.3804	0.0526
		Gestational age at scan^3	0.0000	0.0000	0.001	0.3170	0.0874
		Gestational age at scan*sex	0.0000	0.0000	0.065	-0.1306	0.0591
		Gestational age at scan^2*sex	0.0000	0.0000	0.076	0.1588	0.0743
		Gestational age at scan^3*sex	0.0000	0.0000	0.058	0.2817	0.1241
Cingulate gyrus anterior part right WM	Cubic	Gestational age at scan	-0.0002	0.0000	<0.001	-0.8992	0.0404
		Sex	-0.0001	0.0001	0.367	-0.0284	0.0334
		Gestational age at scan^2	0.0000	0.0000	<0.001	0.4172	0.0542
		Gestational age at scan^3	0.0000	0.0000	<0.001	0.3938	0.0855
		Gestational age at scan*sex	0.0000	0.0000	0.970	0.0035	0.0598
		Gestational age at scan^2*sex	0.0000	0.0000	0.661	0.0527	0.0795
		Gestational age at scan^3*sex	0.0000	0.0000	0.748	0.0657	0.1277
Cingulate gyrus anterior part left WM	Quadratic	Gestational age at scan	-0.0002	0.0000	<0.001	-0.5983	0.0224
		Sex	0.0000	0.0001	0.798	-0.0027	0.0286
		Gestational age at scan^2	0.0000	0.0000	<0.001	0.3357	0.0306
		Gestational age at scan*sex	0.0000	0.0000	0.172	-0.0539	0.0324
		Gestational age at scan^2*sex	0.0000	0.0000	0.772	-0.0178	0.0442
Cingulate gyrus posterior part right WM	Cubic	Gestational age at scan	-0.0001	0.0000	<0.001	-1.0364	0.0661
		Sex	0.0000	0.0001	0.952	0.0720	0.0458
		Gestational age at scan^2	0.0000	0.0000	0.769	-0.0415	0.0880
		Gestational age at scan^3	0.0000	0.0000	0.019	0.3261	0.1197
		Gestational age at scan*sex	0.0000	0.0000	0.056	-0.2081	0.0912
		Gestational age at scan^2*sex	0.0000	0.0000	0.184	0.2061	0.1228

		Gestational age at scan^3*sex	0.0000	0.0000	0.118	0.3228	0.1685
Cingulate gyrus posterior part left WM	Cubic	Gestational age at scan	-0.0001	0.0000	<0.001	-0.9550	0.0607
		Sex	0.0000	0.0001	0.955	0.0648	0.0453
		Gestational age at scan^2	0.0000	0.0000	0.385	-0.1051	0.0885
		Gestational age at scan^3	0.0000	0.0000	0.122	0.2073	0.1093
		Gestational age at scan*sex	0.0000	0.0000	0.041	-0.2093	0.0866
		Gestational age at scan^2*sex	0.0000	0.0000	0.217	0.2012	0.1276
		Gestational age at scan^3*sex	0.0000	0.0000	0.143	0.2884	0.1594
Frontal lobe right WM	Cubic	Gestational age at scan	-0.0024	0.0001	<0.001	-1.1753	0.0287
		Sex	0.0013	0.0004	0.007	0.1220	0.0219
		Gestational age at scan^2	0.0000	0.0000	0.225	-0.0634	0.0408
		Gestational age at scan^3	0.0000	0.0000	<0.001	0.2475	0.0536
		Gestational age at scan*sex	-0.0001	0.0001	0.217	-0.0632	0.0401
		Gestational age at scan^2*sex	0.0000	0.0000	0.445	0.0614	0.0572
		Gestational age at scan^3*sex	0.0000	0.0000	0.367	0.0935	0.0761
Frontal lobe left WM	Cubic	Gestational age at scan	-0.0024	0.0001	<0.001	-1.2295	0.0302
		Sex	0.0011	0.0005	0.043	0.0936	0.0223
		Gestational age at scan^2	0.0000	0.0000	0.027	-0.1141	0.0440
		Gestational age at scan^3	0.0000	0.0000	<0.001	0.2646	0.0548
		Gestational age at scan*sex	-0.0001	0.0001	0.221	-0.0658	0.0420
		Gestational age at scan^2*sex	0.0000	0.0000	0.666	0.0403	0.0617
		Gestational age at scan^3*sex	0.0000	0.0000	0.385	0.0926	0.0777
Parietal lobe right WM	Cubic	Gestational age at scan	-0.0015	0.0001	<0.001	-1.0031	0.0364
		Sex	0.0008	0.0003	0.030	0.0861	0.0269
		Gestational age at scan^2	0.0000	0.0000	0.005	0.1533	0.0477
		Gestational age at scan^3	0.0000	0.0000	0.003	0.2441	0.0729
		Gestational age at scan*sex	-0.0001	0.0001	0.347	-0.0640	0.0503
		Gestational age at scan^2*sex	0.0000	0.0000	0.793	0.0285	0.0667
		Gestational age at scan^3*sex	0.0000	0.0000	0.606	0.0794	0.1023
Parietal lobe left WM	Cubic	Gestational age at scan	-0.0015	0.0001	<0.001	-0.9737	0.0341
		Sex	0.0009	0.0003	0.019	0.0970	0.0267

		Gestational age at scan^2	0.0000	0.0000	0.005	0.1449	0.0452
		Gestational age at scan^3	0.0000	0.0000	0.026	0.1862	0.0714
		Gestational age at scan*sex	-0.0001	0.0001	0.384	-0.0575	0.0482
		Gestational age at scan^2*sex	0.0000	0.0000	0.643	0.0446	0.0641
		Gestational age at scan^3*sex	0.0000	0.0000	0.553	0.0906	0.1020
Thalamus right WM	Linear	Gestational age at scan	0.0000	0.0000	<0.001	0.8178	0.0321
		Sex	0.0000	0.0000	0.016	-0.1159	0.0444
		Gestational age at scan*sex	0.0000	0.0000	0.193	-0.0688	0.0455
Thalamus left WM	Linear	Gestational age at scan	0.0000	0.0000	<0.001	0.5680	0.0445
		Sex	0.0000	0.0000	0.501	-0.0519	0.0625
		Gestational age at scan*sex	0.0000	0.0000	0.003	-0.2079	0.0639
Temporal lobe left WM (merged region)	Linear	Gestational age at scan	-0.0010	0.0000	<0.001	0.8976	0.0231
		Sex	0.0002	0.0002	0.376	-0.0330	0.0300
		Gestational age at scan*sex	-0.0001	0.0000	0.053	0.0330	0.0309
Temporal lobe right WM (merged region)	Linear	Gestational age at scan	-0.0010	0.0000	<0.001	1.0483	0.0237
		Sex	0.0003	0.0002	0.146	0.0022	0.0279
		Gestational age at scan*sex	0.0000	0.0000	0.465	-0.0130	0.0312

Supplementary Table 3 - Absolute Analysis model comparisons

Region	Model type	BIC value
Total Brain Volume	Linear	18577.13
	Quadratic	18549.85
	Cubic	18563.17
Total Gray Matter	Linear	17635.43
	Quadratic	17568.43
	Cubic	17581.62
Total White Matter	Linear	17369.22
	Quadratic	17299.86
	Cubic	17313.03
CSF	Linear	17723.58
	Quadratic	17654.82
	Cubic	17360.74
Total Intracranial Volume	Linear	19003.25
	Quadratic	19011.59
	Cubic	18962.49
Hippocampus left	Linear	9359.00
	Quadratic	9368.76
	Cubic	9380.98
Hippocampus right	Linear	9379.42
	Quadratic	9392.25
	Cubic	9405.49
Amygdala left	Linear	8764.64
	Quadratic	8777.66
	Cubic	8755.19
Amygdala right	Linear	8965.93
	Quadratic	8978.68
	Cubic	8967.34
Anterior temporal lobe medial part left GM	Linear	9906.57
	Quadratic	9862.65
	Cubic	9872.76
Anterior temporal lobe medial part right GM	Linear	9939.14
	Quadratic	9886.61
	Cubic	9891.01
Anterior temporal lobe lateral part left GM	Linear	10104.80
	Quadratic	10033.60
	Cubic	10034.22
Anterior temporal lobe lateral part right GM	Linear	10069.90
	Quadratic	9951.42
	Cubic	9955.28
Gyri parahippocampalis et ambiens anterior part left GM	Linear	10032.94
	Quadratic	10043.60
	Cubic	10026.34
Gyri parahippocampalis et ambiens anterior part right GM	Linear	9912.88
	Quadratic	9902.62
	Cubic	9896.37
Superior temporal gyrus middle part left GM	Linear	11510.24
	Quadratic	11349.15

	Cubic	11359.70
Superior temporal gyrus middle part right GM	Linear	11493.60
	Quadratic	11348.68
	Cubic	11356.56
Medial and inferior temporal gyri anterior part left GM	Linear	11597.92
	Quadratic	11369.74
	Cubic	11377.17
Medial and inferior temporal gyri anterior part right GM	Linear	11645.93
	Quadratic	11355.35
	Cubic	11356.05
Lateral occipitotemporal gyrus fusiformis anterior part left GM	Linear	9711.14
	Quadratic	9709.63
	Cubic	9722.56
Lateral occipitotemporal gyrus fusiformis anterior part right GM	Linear	9860.87
	Quadratic	9828.51
	Cubic	9837.90
Cerebellum left	Linear	13717.96
	Quadratic	13334.67
	Cubic	13347.48
Cerebellum right	Linear	13632.87
	Quadratic	13319.84
	Cubic	13331.34
Brainstem	Linear	11970.12
	Quadratic	11966.58
	Cubic	11968.97
Insula right GM	Linear	10488.62
	Quadratic	10446.36
	Cubic	10457.13
Insula left GM	Linear	10615.92
	Quadratic	10577.87
	Cubic	10590.75
Occipital lobe right GM	Linear	13725.43
	Quadratic	13569.29
	Cubic	13582.10
Occipital lobe left GM	Linear	13720.35
	Quadratic	13524.35
	Cubic	13536.08
Gyri parahippocampalis et ambiens posterior part right GM	Linear	9664.63
	Quadratic	9610.39
	Cubic	9617.47
Gyri parahippocampalis et ambiens posterior part left GM	Linear	9618.44
	Quadratic	9597.91
	Cubic	9602.20
Lateral occipitotemporal gyrus fusiformis posterior part right GM	Linear	10296.00
	Quadratic	10093.92
	Cubic	10098.35
Lateral occipitotemporal gyrus fusiformis posterior part left GM	Linear	10267.97
	Quadratic	10161.05

	Cubic	10162.86
Medial and inferior temporal gyri posterior part right GM	Linear	12122.08
	Quadratic	11849.99
	Cubic	11861.82
Medial and inferior temporal gyri posterior part left GM	Linear	12228.07
	Quadratic	11926.01
	Cubic	11938.73
Superior temporal gyrus posterior part right GM	Linear	10495.64
	Quadratic	10343.52
	Cubic	10355.57
Superior temporal gyrus posterior part left GM	Linear	10315.18
	Quadratic	10192.09
	Cubic	10201.79
Cingulate gyrus anterior part right GM	Linear	10748.19
	Quadratic	10703.15
	Cubic	10708.41
Cingulate gyrus anterior part left GM	Linear	10801.26
	Quadratic	10805.23
	Cubic	10797.85
Cingulate gyrus posterior part right GM	Linear	10965.37
	Quadratic	10873.24
	Cubic	10885.92
Cingulate gyrus posterior part left GM	Linear	10868.49
	Quadratic	10769.54
	Cubic	10782.89
Frontal lobe right GM	Linear	14774.01
	Quadratic	14591.84
	Cubic	14599.68
Frontal lobe left GM	Linear	14776.86
	Quadratic	14583.25
	Cubic	14589.82
Parietal lobe right GM	Linear	14251.02
	Quadratic	13957.67
	Cubic	13968.59
Parietal lobe left GM	Linear	14277.54
	Quadratic	13974.06
	Cubic	13984.75
Temporal lobe right GM (merged region)	Linear	13130.00
	Quadratic	12576.00
	Cubic	12588.00
Temporal lobe left GM (merged region)	Linear	12634.00
	Quadratic	12364.00
	Cubic	12374.00
Caudate nucleus right	Linear	10826.77
	Quadratic	10810.73
	Cubic	10779.49
Caudate nucleus left	Linear	10830.20
	Quadratic	10804.25

	Cubic	10779.63
Thalamus right	Linear	11498.46
	Quadratic	11416.62
	Cubic	11284.46
Thalamus left	Linear	11461.15
	Quadratic	11360.88
	Cubic	11240.23
Subthalamic nucleus right	Linear	7733.47
	Quadratic	7639.84
	Cubic	7468.41
Subthalamic nucleus left	Linear	7727.92
	Quadratic	7591.61
	Cubic	7445.59
Lentiform nucleus right	Linear	11427.66
	Quadratic	11416.47
	Cubic	11426.61
Lentiform nucleus left	Linear	11401.15
	Quadratic	11406.12
	Cubic	11405.85
Corpus Callosum	Linear	11808.72
	Quadratic	11681.94
	Cubic	11685.64
Lateral Ventricle left	Linear	13239.28
	Quadratic	13223.74
	Cubic	13195.08
Lateral Ventricle right	Linear	12835.03
	Quadratic	12824.15
	Cubic	12799.35
Anterior temporal lobe medial part left WM	Linear	9763.17
	Quadratic	9732.70
	Cubic	9733.57
Anterior temporal lobe medial part right WM	Linear	9767.63
	Quadratic	9753.42
	Cubic	9759.30
Anterior temporal lobe lateral part left WM	Linear	9544.87
	Quadratic	9469.63
	Cubic	9481.79
Anterior temporal lobe lateral part right WM	Linear	9502.24
	Quadratic	9433.23
	Cubic	9446.03
Gyri parahippocampalis et ambiens anterior part left WM	Linear	9419.25
	Quadratic	9405.63
	Cubic	9392.31
Gyri parahippocampalis et ambiens anterior part right WM	Linear	9200.57
	Quadratic	9201.05
	Cubic	9187.75
Superior temporal gyrus middle part left WM	Linear	11496.16
	Quadratic	11439.40

	Cubic	11451.79
Superior temporal gyrus middle part right WM	Linear	11258.40
	Quadratic	11226.69
	Cubic	11235.77
Medial and inferior temporal gyri anterior part left WM	Linear	11662.10
	Quadratic	11608.28
	Cubic	11614.39
Medial and inferior temporal gyri anterior part right WM	Linear	11568.62
	Quadratic	11512.53
	Cubic	11520.29
Lateral occipitotemporal gyrus fusiformis anterior part left WM	Linear	10040.71
	Quadratic	10014.50
	Cubic	10007.86
Lateral occipitotemporal gyrus fusiformis anterior part right WM	Linear	9956.88
	Quadratic	9958.04
	Cubic	9967.05
Insula right WM	Linear	11334.16
	Quadratic	11314.88
	Cubic	11327.40
Insula left WM	Linear	11367.44
	Quadratic	11339.57
	Cubic	11351.77
Occipital lobe right WM	Linear	13164.04
	Quadratic	13117.01
	Cubic	13128.31
Occipital lobe left WM	Linear	13131.61
	Quadratic	13122.08
	Cubic	13133.59
Gyri parahippocampalis et ambiens posterior part right WM	Linear	9347.35
	Quadratic	9351.93
	Cubic	9364.61
Gyri parahippocampalis et ambiens posterior part left WM	Linear	9452.41
	Quadratic	9457.88
	Cubic	9470.73
Lateral occipitotemporal gyrus fusiformis posterior part right WM	Linear	10439.39
	Quadratic	10439.61
	Cubic	10409.95
Lateral occipitotemporal gyrus fusiformis posterior part left WM	Linear	10353.44
	Quadratic	10366.12
	Cubic	10346.27
Medial and inferior temporal gyri posterior part right WM	Linear	12070.52
	Quadratic	11989.32
	Cubic	11995.13
Medial and inferior temporal gyri posterior part left WM	Linear	12056.18
	Quadratic	11966.71
	Cubic	11976.67
Superior temporal gyrus posterior part right WM	Linear	10774.09
	Quadratic	10782.56

	Cubic	10718.47
Superior temporal gyrus posterior part left WM	Linear	10534.60
	Quadratic	10494.97
	Cubic	10430.65
Cingulate gyrus anterior part right WM	Linear	10715.70
	Quadratic	10700.13
	Cubic	10673.80
Cingulate gyrus anterior part left WM	Linear	10835.58
	Quadratic	10823.57
	Cubic	10827.91
Cingulate gyrus posterior part right WM	Linear	10401.50
	Quadratic	10353.81
	Cubic	10361.75
Cingulate gyrus posterior part left WM	Linear	10470.41
	Quadratic	10417.33
	Cubic	10425.26
Frontal lobe right WM	Linear	14893.65
	Quadratic	14806.61
	Cubic	14816.86
Frontal lobe left WM	Linear	14907.12
	Quadratic	14819.13
	Cubic	14829.14
Parietal lobe right WM	Linear	14071.56
	Quadratic	14007.52
	Cubic	14016.66
Parietal lobe left WM	Linear	14084.05
	Quadratic	14014.97
	Cubic	14023.91
Thalamus right WM	Linear	8864.15
	Quadratic	8708.04
	Cubic	8717.89
Thalamus left WM	Linear	8470.30
	Quadratic	8437.45
	Cubic	8448.84
Temporal lobe left WM (merged region)	Linear	13645.00
	Quadratic	13609.00
	Cubic	13620.00
Temporal lobe right WM (merged region)	Linear	13654.00
	Quadratic	13636.00
	Cubic	13647.00

Supplementary Table 4 - Proportional analysis model comparisons

Region	Model type	BIC value
Total Gray Matter	Linear	-4308.49
	Quadratic	-4479.66
	Cubic	-4659.48
Total White Matter	Linear	-4308.49
	Quadratic	-4479.66
	Cubic	-4659.48
Hippocampus left	Linear	-10436.09
	Quadratic	-10480.12
	Cubic	-10483.14
Hippocampus right	Linear	-10427.00
	Quadratic	-10451.98
	Cubic	-10442.61
Amygdala left	Linear	-10757.15
	Quadratic	-10819.70
	Cubic	-10809.06
Amygdala right	Linear	-10604.45
	Quadratic	-10652.70
	Cubic	-10640.06
Anterior temporal lobe medial part left GM	Linear	-10580.73
	Quadratic	-10591.89
	Cubic	-10579.17
Anterior temporal lobe medial part right GM	Linear	-10622.85
	Quadratic	-10630.01
	Cubic	-10628.50
Anterior temporal lobe lateral part left GM	Linear	-10480.39
	Quadratic	-10491.52
	Cubic	-10482.90
Anterior temporal lobe lateral part right GM	Linear	-10543.08
	Quadratic	-10582.87
	Cubic	-10572.48
Gyri parahippocampalis et ambiens anterior part left GM	Linear	-10115.29
	Quadratic	-10110.04
	Cubic	-10136.33
Gyri parahippocampalis et ambiens anterior part right GM	Linear	-10243.84
	Quadratic	-10244.98
	Cubic	-10296.69
Superior temporal gyrus middle part left GM	Linear	-9329.34
	Quadratic	-9328.32
	Cubic	-9333.93
Superior temporal gyrus middle part right GM	Linear	-9375.67
	Quadratic	-9370.58
	Cubic	-9393.72
Medial and inferior temporal gyri anterior part left GM	Linear	-9099.88
	Quadratic	-9393.29
	Cubic	-9415.93
Medial and inferior temporal gyri anterior part right GM	Linear	-9095.32
	Quadratic	-9424.29

	Cubic	-9436.75
Lateral occipitotemporal gyrus fusiformis anterior part left GM	Linear	-10721.21
	Quadratic	-10746.34
	Cubic	-10733.09
Lateral occipitotemporal gyrus fusiformis anterior part right GM	Linear	-10652.45
	Quadratic	-10648.28
	Cubic	-10635.53
Cerebellum left	Linear	-7310.16
	Quadratic	-7305.51
	Cubic	-7307.08
Cerebellum right	Linear	-7298.91
	Quadratic	-7286.06
	Cubic	-7278.04
Brainstem	Linear	-7746.55
	Quadratic	-8195.74
	Cubic	-8227.27
Insula right GM	Linear	-10082.19
	Quadratic	-10236.18
	Cubic	-10261.52
Insula left GM	Linear	-10015.07
	Quadratic	-10169.22
	Cubic	-10162.21
Occipital lobe right GM	Linear	-7329.92
	Quadratic	-7319.39
	Cubic	-7320.10
Occipital lobe left GM	Linear	-7450.56
	Quadratic	-7454.38
	Cubic	-7459.90
Gyri parahippocampalis et ambiens posterior part right GM	Linear	-10748.82
	Quadratic	-10743.08
	Cubic	-10792.86
Gyri parahippocampalis et ambiens posterior part left GM	Linear	-10753.12
	Quadratic	-10744.43
	Cubic	-10764.95
Lateral occipitotemporal gyrus fusiformis posterior part right GM	Linear	-10317.71
	Quadratic	-10346.61
	Cubic	-10448.58
Lateral occipitotemporal gyrus fusiformis posterior part left GM	Linear	-10276.71
	Quadratic	-10266.31
	Cubic	-10328.94
Medial and inferior temporal gyri posterior part right GM	Linear	-8766.21
	Quadratic	-9015.72
	Cubic	-9076.83
Medial and inferior temporal gyri posterior part left GM	Linear	-8495.67
	Quadratic	-8804.85
	Cubic	-8893.68
Superior temporal gyrus posterior part right GM	Linear	-10262.53
	Quadratic	-10252.63

	Cubic	-10255.39
Superior temporal gyrus posterior part left GM	Linear	-10347.72
	Quadratic	-10357.11
	Cubic	-10351.71
Cingulate gyrus anterior part right GM	Linear	-9821.58
	Quadratic	-9814.79
	Cubic	-9818.96
Cingulate gyrus anterior part left GM	Linear	-9612.89
	Quadratic	-9611.37
	Cubic	-9612.46
Cingulate gyrus posterior part right GM	Linear	-9789.89
	Quadratic	-9790.71
	Cubic	-9782.60
Cingulate gyrus posterior part left GM	Linear	-9866.30
	Quadratic	-9854.38
	Cubic	-9841.89
Frontal lobe right GM	Linear	-6925.13
	Quadratic	-6917.17
	Cubic	-6904.00
Frontal lobe left GM	Linear	-6919.45
	Quadratic	-6922.28
	Cubic	-6909.54
Parietal lobe right GM	Linear	-7330.10
	Quadratic	-7404.77
	Cubic	-7437.65
Parietal lobe left GM	Linear	-7330.13
	Quadratic	-7419.08
	Cubic	-7444.17
Temporal lobe right GM (merged region)	Linear	-7288.79
	Quadratic	-7483.70
	Cubic	-7596.76
Temporal lobe left GM (merged region)	Linear	-7774.27
	Quadratic	-7791.10
	Cubic	-7842.34
Caudate nucleus right	Linear	-9537.55
	Quadratic	-9525.71
	Cubic	-9600.98
Caudate nucleus left	Linear	-9607.47
	Quadratic	-9594.30
	Cubic	-9671.10
Thalamus right	Linear	-8375.96
	Quadratic	-8469.83
	Cubic	-9043.75
Thalamus left	Linear	-8298.41
	Quadratic	-8447.91
	Cubic	-9046.75
Subthalamic nucleus right	Linear	-11336.48
	Quadratic	-11373.62

	Cubic	-11658.85
Subthalamic nucleus left	Linear	-11193.28
	Quadratic	-11201.86
	Cubic	-11404.68
Lentiform nucleus right	Linear	-8526.28
	Quadratic	-8628.52
	Cubic	-8718.79
Lentiform nucleus left	Linear	-8655.68
	Quadratic	-8682.91
	Cubic	-8774.53
Corpus Callosum	Linear	-7990.04
	Quadratic	-8324.80
	Cubic	-8545.47
Lateral Ventricle left	Linear	-6310.30
	Quadratic	-6353.68
	Cubic	-6353.42
Lateral Ventricle right	Linear	-6641.84
	Quadratic	-6715.37
	Cubic	-6711.47
Anterior temporal lobe medial part left WM	Linear	-10530.24
	Quadratic	-10529.51
	Cubic	-10530.13
Anterior temporal lobe medial part right WM	Linear	-10577.22
	Quadratic	-10574.60
	Cubic	-10568.45
Anterior temporal lobe lateral part left WM	Linear	-11090.76
	Quadratic	-11078.22
	Cubic	-11066.14
Anterior temporal lobe lateral part right WM	Linear	-11126.51
	Quadratic	-11114.50
	Cubic	-11101.49
Gyri parahippocampalis et ambiens anterior part left WM	Linear	-10520.11
	Quadratic	-10522.67
	Cubic	-10548.70
Gyri parahippocampalis et ambiens anterior part right WM	Linear	-10816.02
	Quadratic	-10803.03
	Cubic	-10830.62
Superior temporal gyrus middle part left WM	Linear	-8742.51
	Quadratic	-8806.90
	Cubic	-8864.46
Superior temporal gyrus middle part right WM	Linear	-9101.38
	Quadratic	-9090.65
	Cubic	-9103.92
Medial and inferior temporal gyri anterior part left WM	Linear	-8615.27
	Quadratic	-8620.67
	Cubic	-8614.43
Medial and inferior temporal gyri anterior part right WM	Linear	-8697.97
	Quadratic	-8717.37

	Cubic	-8706.74
Lateral occipitotemporal gyrus fusiformis anterior part left WM	Linear	-9912.79
	Quadratic	-9971.71
	Cubic	-10027.05
Lateral occipitotemporal gyrus fusiformis anterior part right WM	Linear	-10040.37
	Quadratic	-10073.11
	Cubic	-10083.46
Insula right WM	Linear	-8890.49
	Quadratic	-9038.04
	Cubic	-9035.97
Insula left WM	Linear	-8763.00
	Quadratic	-8934.43
	Cubic	-8937.45
Occipital lobe right WM	Linear	-7180.47
	Quadratic	-7380.11
	Cubic	-7478.90
Occipital lobe left WM	Linear	-7343.45
	Quadratic	-7478.91
	Cubic	-7520.37
Gyri parahippocampalis et ambiens posterior part right WM	Linear	-10819.27
	Quadratic	-10818.67
	Cubic	-10808.89
Gyri parahippocampalis et ambiens posterior part left WM	Linear	-10742.57
	Quadratic	-10734.39
	Cubic	-10722.15
Lateral occipitotemporal gyrus fusiformis posterior part right WM	Linear	-9898.43
	Quadratic	-9886.15
	Cubic	-10001.11
Lateral occipitotemporal gyrus fusiformis posterior part left WM	Linear	-9989.85
	Quadratic	-10003.94
	Cubic	-10063.12
Medial and inferior temporal gyri posterior part right WM	Linear	-8495.87
	Quadratic	-8531.80
	Cubic	-8523.33
Medial and inferior temporal gyri posterior part left WM	Linear	-8410.88
	Quadratic	-8400.96
	Cubic	-8410.92
Superior temporal gyrus posterior part right WM	Linear	-8881.14
	Quadratic	-9135.65
	Cubic	-9166.91
Superior temporal gyrus posterior part left WM	Linear	-9286.37
	Quadratic	-9309.92
	Cubic	-9370.28
Cingulate gyrus anterior part right WM	Linear	-9380.13
	Quadratic	-9433.02
	Cubic	-9446.71
Cingulate gyrus anterior part left WM	Linear	-9005.27
	Quadratic	-9211.53

	Cubic	-9199.58
Cingulate gyrus posterior part right WM	Linear	-9885.57
	Quadratic	-9917.44
	Cubic	-9930.51
Cingulate gyrus posterior part left WM	Linear	-9820.54
	Quadratic	-9852.93
	Cubic	-9860.63
Frontal lobe right WM	Linear	-6340.76
	Quadratic	-6531.76
	Cubic	-6591.67
Frontal lobe left WM	Linear	-6302.13
	Quadratic	-6553.74
	Cubic	-6604.94
Parietal lobe right WM	Linear	-6673.82
	Quadratic	-6685.29
	Cubic	-6813.63
Parietal lobe left WM	Linear	-6660.33
	Quadratic	-6665.71
	Cubic	-6793.11
Thalamus right WM	Linear	-11483.47
	Quadratic	-11473.41
	Cubic	-11463.19
Thalamus left WM	Linear	-11632.32
	Quadratic	-11627.07
	Cubic	-11619.34
Temporal lobe left WM (merged region)	Linear	-7257.75
	Quadratic	-7245.43
	Cubic	-7244.62
Temporal lobe right WM (merged region)	Linear	-23389.89
	Quadratic	-24550.01
	Cubic	-25382.46

Supplementary Table 5 - Extended Model Comparisons

Region	Model	BIC
Total Brain Volume	Quadratic	18587.95
	Cubic	18600.26
	Linear	18606.77
	Logarithmic	18758.71
	Exponential	18875.90
Total Gray Matter	Quadratic	17591.26
	Cubic	17603.14
	Linear	17652.80
	Exponential	17819.71
	Logarithmic	17833.57
Total White Matter	Logarithmic	17333.37
	Quadratic	17343.20
	Cubic	17356.43
	Linear	17392.36
	Exponential	17747.02
CSF	Cubic	17364.08
	Quadratic	17661.91
	Logarithmic	17698.68
	Linear	17717.21
	Exponential	17719.34
ICV	Cubic	18988.91
	Linear	19036.85
	Quadratic	19048.67
	Logarithmic	19068.59
	Exponential	19245.86
Hippocampus left	Linear	9377.09
	Logarithmic	9384.32
	Quadratic	9386.25
	Cubic	9398.18
	Exponential	9572.10
Hippocampus right	Linear	9394.97
	Quadratic	9406.87

	Cubic	9420.18
	Logarithmic	9420.62
	Exponential	9616.04
Amygdala left	Cubic	8750.82
	Linear	8759.02
	Quadratic	8772.05
	Logarithmic	8794.47
	Exponential	9025.22
Amygdala right	Linear	8962.54
	Cubic	8964.69
	Quadratic	8975.43
	Logarithmic	9009.71
	Exponential	9195.88
Anterior temporal lobe medial part left GM	Quadratic	9878.42
	Cubic	9886.71
	Linear	9909.48
	Exponential	9945.11
	Logarithmic	9994.76
Anterior temporal lobe medial part right GM	Quadratic	9904.32
	Cubic	9905.46
	Linear	9946.89
	Exponential	9959.06
	Logarithmic	10041.69
Anterior temporal lobe lateral part left GM	Quadratic	10042.84
	Cubic	10043.70
	Exponential	10097.46
	Linear	10107.44
	Logarithmic	10209.17
Anterior temporal lobe lateral part right GM	Quadratic	9952.45
	Cubic	9956.31
	Exponential	9990.87
	Linear	10065.25
	Logarithmic	10198.45

Gyri parahippocampalis et ambiens anterior part left GM	Cubic	10022.57
	Linear	10027.59
	Quadratic	10039.12
	Logarithmic	10077.22
	Exponential	10216.34
Gyri parahippocampalis et ambiens anterior part right GM	Cubic	9895.76
	Quadratic	9902.29
	Linear	9909.22
	Logarithmic	9994.09
	Exponential	10019.17
Superior temporal gyrus middle part left GM	Quadratic	11351.36
	Cubic	11361.73
	Linear	11503.72
	Exponential	11559.83
	Logarithmic	11698.94
Superior temporal gyrus middle part right GM	Quadratic	11351.73
	Cubic	11359.69
	Linear	11488.23
	Exponential	11552.31
	Logarithmic	11680.98
Medial and inferior temporal gyri anterior part left GM	Quadratic	11376.41
	Cubic	11383.40
	Exponential	11419.57
	Linear	11591.36
	Logarithmic	11745.70
Medial and inferior temporal gyri anterior part right GM	Quadratic	11356.98
	Cubic	11358.06
	Exponential	11394.42
	Linear	11639.26
	Logarithmic	11819.02
Lateral occipitotemporal gyrus fusiformis anterior part left GM	Quadratic	9708.44
	Linear	9709.97
	Cubic	9720.76

	Logarithmic	9768.71
	Exponential	9795.93
Lateral occipitotemporal gyrus fusiformis anterior part right GM	Quadratic	9831.81
	Cubic	9840.07
	Linear	9864.83
	Exponential	9886.92
	Logarithmic	9946.83
Cerebellum left	Quadratic	13339.29
	Cubic	13352.00
	Exponential	13529.42
	Linear	13711.28
	Logarithmic	13994.37
Cerebellum right	Quadratic	13324.26
	Cubic	13335.63
	Exponential	13504.12
	Linear	13626.68
	Logarithmic	13896.72
Brainstem	Linear	11992.59
	Quadratic	11995.43
	Cubic	11995.84
	Logarithmic	11999.86
	Exponential	12210.53
Insula right GM	Quadratic	10473.43
	Cubic	10483.65
	Linear	10511.93
	Logarithmic	10648.52
	Exponential	10688.23
Insula left GM	Quadratic	10590.99
	Cubic	10604.08
	Linear	10627.31
	Logarithmic	10768.69
	Exponential	10848.89
Occipital lobe right GM	Quadratic	13578.82

	Cubic	13590.31
	Exponential	13702.87
	Linear	13721.58
	Logarithmic	13905.16
Occipital lobe left GM	Quadratic	13533.20
	Cubic	13543.96
	Exponential	13652.28
	Linear	13714.70
	Logarithmic	13916.95
Gyri parahippocampalis et ambiens posterior part right GM	Quadratic	9607.12
	Cubic	9614.27
	Linear	9658.90
	Exponential	9705.07
	Logarithmic	9778.98
Gyri parahippocampalis et ambiens posterior part left GM	Quadratic	9595.24
	Cubic	9601.34
	Linear	9612.87
	Exponential	9702.09
	Logarithmic	9708.30
Lateral occipitotemporal gyrus fusiformis posterior part right GM	Quadratic	10089.59
	Cubic	10094.63
	Exponential	10195.90
	Linear	10289.32
	Logarithmic	10452.83
Lateral occipitotemporal gyrus fusiformis posterior part left GM	Quadratic	10154.86
	Cubic	10157.81
	Exponential	10254.85
	Linear	10261.29
	Logarithmic	10389.64
Medial and inferior temporal gyri posterior part right GM	Quadratic	11866.42
	Cubic	11877.42
	Exponential	11996.01
	Linear	12117.93

	Logarithmic	12311.77
Medial and inferior temporal gyri posterior part left GM	Quadratic	11932.36
	Cubic	11944.99
	Exponential	12048.92
	Linear	12221.39
	Logarithmic	12416.99
Superior temporal gyrus posterior part right GM	Quadratic	10347.64
	Cubic	10358.87
	Exponential	10452.97
	Linear	10493.35
	Logarithmic	10647.77
Superior temporal gyrus posterior part left GM	Quadratic	10197.12
	Cubic	10206.28
	Exponential	10301.75
	Linear	10313.51
	Logarithmic	10455.95
Cingulate gyrus anterior part right GM	Quadratic	10717.44
	Cubic	10723.28
	Linear	10747.64
	Exponential	10827.02
	Logarithmic	10839.73
Cingulate gyrus anterior part left GM	Cubic	10806.00
	Linear	10806.08
	Quadratic	10813.50
	Logarithmic	10845.20
	Exponential	10902.60
Cingulate gyrus posterior part right GM	Quadratic	10876.57
	Cubic	10889.33
	Linear	10960.69
	Exponential	10992.07
	Logarithmic	11083.70
Cingulate gyrus posterior part left GM	Quadratic	10778.40
	Cubic	10791.74

	Linear	10867.32
	Exponential	10890.53
	Logarithmic	11000.13
Frontal lobe right GM	Quadratic	14620.82
	Cubic	14624.66
	Linear	14779.63
	Exponential	14800.86
	Logarithmic	14986.03
Frontal lobe left GM	Quadratic	14613.76
	Cubic	14615.79
	Exponential	14773.05
	Linear	14782.46
	Logarithmic	14990.82
Parietal lobe right GM	Quadratic	13980.97
	Cubic	13991.23
	Exponential	14125.36
	Linear	14249.81
	Logarithmic	14497.87
Parietal lobe left GM	Quadratic	13989.43
	Cubic	13998.58
	Exponential	14139.11
	Linear	14272.84
	Logarithmic	14522.67
Temporal lobe right GM (merged region)	Quadratic	13688.85
	Cubic	13820.51
	Linear	14035.70
	Logarithmic	14328.74
	Exponential	15902.00
Temporal lobe left GM (merged region)	Quadratic	13227.03
	Cubic	13346.41
	Linear	13480.47
	Logarithmic	13756.83
	Exponential	15476.46

Caudate nucleus right	Cubic	10781.72
	Quadratic	10809.08
	Linear	10821.01
	Logarithmic	10928.26
	Exponential	11001.49
Caudate nucleus left	Cubic	10783.91
	Quadratic	10805.45
	Linear	10824.61
	Logarithmic	10934.55
	Exponential	10971.04
Thalamus right	Cubic	11283.50
	Quadratic	11410.49
	Linear	11491.78
	Exponential	11711.29
	Logarithmic	11723.77
Thalamus left	Cubic	11244.10
	Quadratic	11357.39
	Linear	11454.49
	Exponential	11637.79
	Logarithmic	11692.27
Subthalamic nucleus right	Cubic	7466.16
	Quadratic	7637.24
	Logarithmic	7665.61
	Linear	7727.34
	Exponential	7822.81
Subthalamic nucleus left	Cubic	7441.12
	Quadratic	7588.70
	Logarithmic	7671.80
	Linear	7721.24
	Exponential	7768.36
Lentiform nucleus right	Quadratic	11425.64
	Linear	11436.99
	Cubic	11438.11

	Logarithmic	11535.43
	Exponential	11538.79
Lentiform nucleus left	Linear	11413.35
	Quadratic	11417.42
	Cubic	11423.44
	Logarithmic	11502.11
	Exponential	11608.48
Corpus Callosum	Quadratic	11677.18
	Cubic	11680.70
	Exponential	11709.83
	Linear	11802.04
	Logarithmic	11927.52
Lateral Ventricle left	Cubic	13247.62
	Logarithmic	13281.00
	Exponential	13281.23
	Linear	13284.14
	Quadratic	13290.78
Lateral Ventricle right	Cubic	12838.64
	Logarithmic	12863.95
	Linear	12868.44
	Quadratic	12870.10
	Exponential	12878.61
Anterior temporal lobe medial part left WM	Quadratic	9728.80
	Cubic	9730.26
	Linear	9756.49
	Exponential	9784.43
	Logarithmic	9826.24
Anterior temporal lobe medial part right WM	Quadratic	9752.96
	Cubic	9758.55
	Linear	9762.30
	Exponential	9796.46
	Logarithmic	9812.58
Anterior temporal lobe lateral part left WM	Quadratic	9478.63

	Cubic	9491.02
	Exponential	9536.25
	Linear	9545.24
	Logarithmic	9644.49
Anterior temporal lobe lateral part right WM	Quadratic	9454.26
	Cubic	9466.94
	Exponential	9480.13
	Linear	9508.76
	Logarithmic	9599.12
Gyri parahippocampalis et ambiens anterior part left WM	Cubic	9387.27
	Quadratic	9401.17
	Linear	9412.70
	Exponential	9477.97
	Logarithmic	9480.80
Gyri parahippocampalis et ambiens anterior part right WM	Cubic	9181.09
	Linear	9193.88
	Quadratic	9194.37
	Logarithmic	9252.22
	Exponential	9298.76
Superior temporal gyrus middle part left WM	Logarithmic	11456.58
	Quadratic	11456.61
	Cubic	11467.91
	Linear	11506.65
	Exponential	11702.85
Superior temporal gyrus middle part right WM	Logarithmic	11232.94
	Quadratic	11244.50
	Cubic	11251.51
	Linear	11266.53
	Exponential	11451.13
Medial and inferior temporal gyri anterior part left WM	Quadratic	11623.37
	Logarithmic	11624.67
	Cubic	11629.70
	Linear	11667.99

	Exponential	11851.17
Medial and inferior temporal gyri anterior part right WM	Logarithmic	11528.14
	Quadratic	11528.26
	Cubic	11538.65
	Linear	11576.26
	Exponential	11795.10
Lateral occipitotemporal gyrus fusiformis anterior part left WM	Cubic	10010.96
	Quadratic	10015.38
	Linear	10036.23
	Exponential	10076.27
	Logarithmic	10097.80
Lateral occipitotemporal gyrus fusiformis anterior part right WM	Linear	9971.36
	Quadratic	9976.00
	Cubic	9986.02
	Logarithmic	10010.26
	Exponential	10059.73
Insula right WM	Logarithmic	11315.71
	Quadratic	11324.90
	Cubic	11337.61
	Linear	11339.41
	Exponential	11622.48
Insula left WM	Logarithmic	11333.59
	Quadratic	11343.35
	Cubic	11355.69
	Linear	11368.10
	Exponential	11644.94
Occipital lobe right WM	Logarithmic	13152.66
	Quadratic	13160.32
	Cubic	13165.73
	Linear	13193.22
	Exponential	13404.46
Occipital lobe left WM	Logarithmic	13137.26
	Quadratic	13151.22

	Linear	13154.84
	Cubic	13160.64
	Exponential	13384.86
Gyri parahippocampalis et ambiens posterior part right WM	Linear	9355.24
	Logarithmic	9356.93
	Quadratic	9361.60
	Cubic	9374.54
	Exponential	9449.28
Gyri parahippocampalis et ambiens posterior part left WM	Logarithmic	9463.03
	Linear	9466.74
	Quadratic	9473.14
	Cubic	9486.47
	Exponential	9519.20
Lateral occipitotemporal gyrus fusiformis posterior part right WM	Cubic	10411.78
	Linear	10438.62
	Quadratic	10442.78
	Logarithmic	10493.94
	Exponential	10578.18
Lateral occipitotemporal gyrus fusiformis posterior part left WM	Cubic	10351.10
	Linear	10356.07
	Quadratic	10368.62
	Logarithmic	10376.36
	Exponential	10532.62
Medial and inferior temporal gyri posterior part right WM	Quadratic	12026.77
	Cubic	12037.08
	Logarithmic	12039.31
	Linear	12094.66
	Exponential	12328.08
Medial and inferior temporal gyri posterior part left WM	Quadratic	12018.93
	Cubic	12026.59
	Logarithmic	12032.16
	Linear	12081.18
	Exponential	12229.18

Superior temporal gyrus posterior part right WM	Cubic	10750.04
	Logarithmic	10782.32
	Linear	10788.54
	Quadratic	10799.58
	Exponential	10844.62
Superior temporal gyrus posterior part left WM	Cubic	10450.95
	Logarithmic	10511.99
	Quadratic	10519.10
	Linear	10540.13
	Exponential	10598.44
Cingulate gyrus anterior part right WM	Cubic	10697.40
	Logarithmic	10714.83
	Quadratic	10729.52
	Linear	10733.71
	Exponential	10778.76
Cingulate gyrus anterior part left WM	Logarithmic	10848.54
	Quadratic	10860.15
	Linear	10862.47
	Cubic	10864.93
	Exponential	10891.85
Cingulate gyrus posterior part right WM	Logarithmic	10358.66
	Quadratic	10365.87
	Cubic	10372.44
	Linear	10402.52
	Exponential	10587.87
Cingulate gyrus posterior part left WM	Logarithmic	10430.83
	Quadratic	10436.92
	Cubic	10441.27
	Linear	10474.09
	Exponential	10640.46
Frontal lobe right WM	Quadratic	14842.46
	Logarithmic	14842.71
	Cubic	14854.99

	Linear	14912.32
	Exponential	15250.33
Frontal lobe left WM	Quadratic	14849.56
	Logarithmic	14850.49
	Cubic	14861.92
	Linear	14921.29
	Exponential	15259.76
Parietal lobe right WM	Logarithmic	14038.70
	Quadratic	14050.18
	Cubic	14059.54
	Linear	14092.32
	Exponential	14370.01
Parietal lobe left WM	Logarithmic	14045.94
	Quadratic	14056.73
	Cubic	14065.96
	Linear	14100.70
	Exponential	14383.50
Thalamus right WM	Quadratic	8705.87
	Cubic	8715.24
	Exponential	8758.28
	Linear	8857.47
	Logarithmic	9011.13
Thalamus left WM	Quadratic	8431.80
	Cubic	8443.03
	Linear	8464.34
	Exponential	8506.77
	Logarithmic	8566.43
Temporal lobe right WM (merged region)	Quadratic	13550.05
	Linear	13621.31
	Cubic	13688.40
	Logarithmic	13721.45
	Exponential	15499.09
Temporal lobe left WM (merged region)	Quadratic	13523.12

Linear	13612.68
Cubic	13643.29
Logarithmic	13671.41
Exponential	15432.11

Supplementary Table 6 - Sample breakdown by age and sex

Postconceptional age (weeks)	No. females	No. males
21-22	3	2
22-23	5	3
23-24	3	6
24-25	8	13
25-26	15	8
26-27	8	9
27-28	8	14
28-29	13	12
29-30	9	13
30-31	6	11
31-32	12	11
32-33	11	12
33-34	7	10
34-35	4	5
35-36	6	3
36-37	4	5
37-38	4	3
38-39	13	21
39-40	27	40
40-41	40	60
41-42	57	58
42-43	39	49
43-44	47	44
44-45	21	16

Supplementary Figure 1. Global brain volumes after accounting for postnatal age at scan

Brain volumes in mm³, with fitted values regressed for postnatal age at scan for neonatal scans, plotted against postconceptional age in weeks.

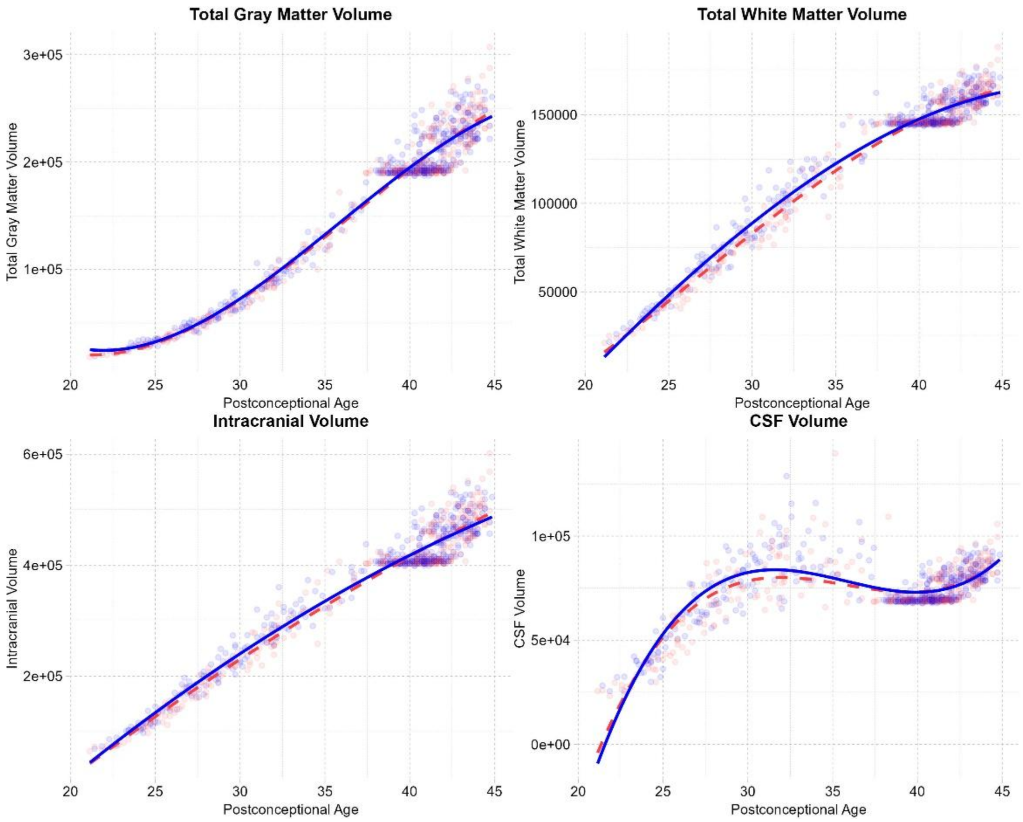

Supplementary Figure 2. Subcortical brain volumes after accounting for postnatal age at scan

Brain volumes in mm³, with fitted values regressed for postnatal age at scan for neonatal scans, plotted against postconceptional age in weeks.

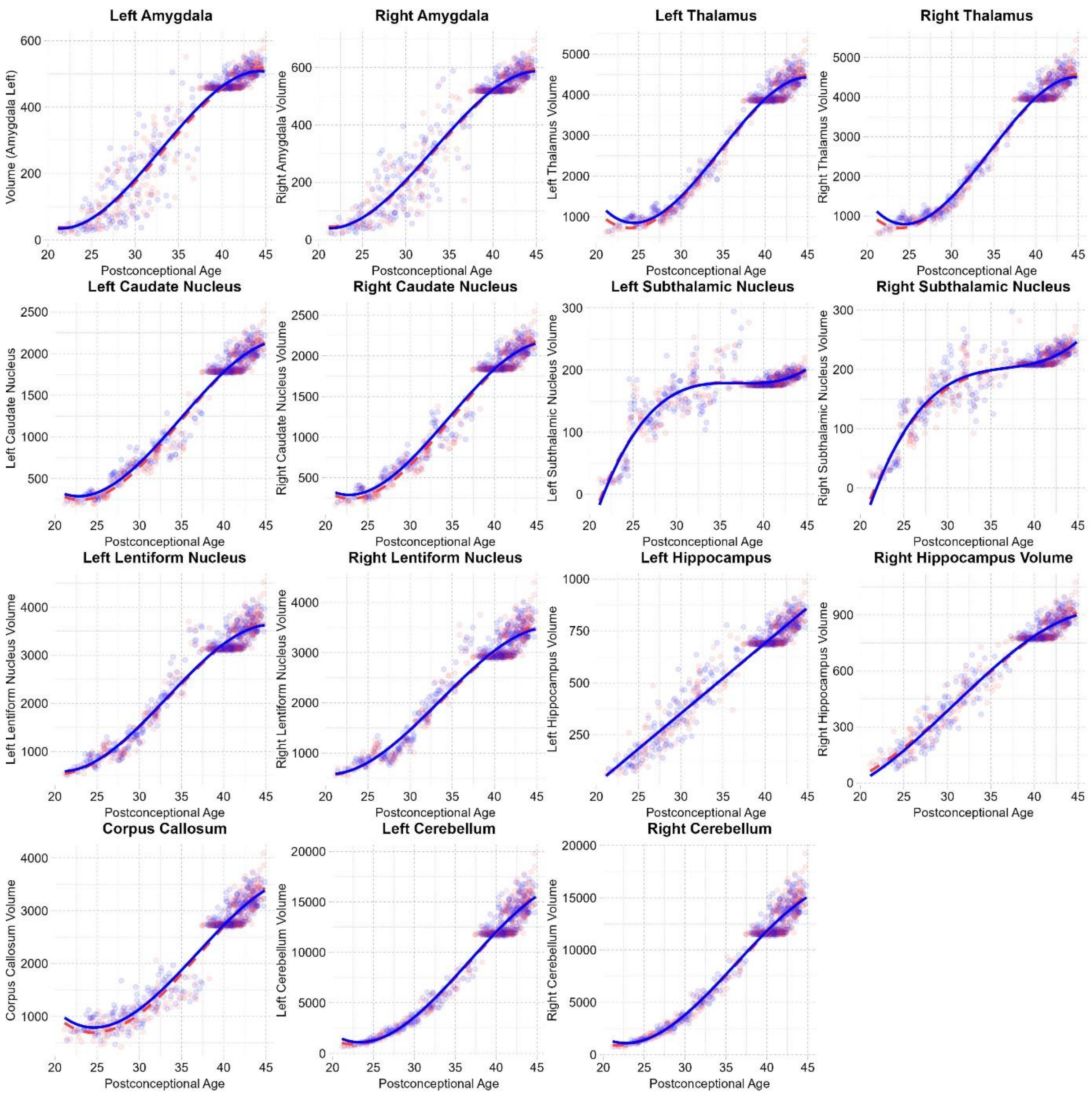

Supplementary Figure 3. Total brain volume postnatal growth

To further pinpoint the exact timing that the postnatal deceleration emerges, Figure 4 plots residualised total brain volume (regressed for postconceptional age) against postnatal age at scan for neonatal scans only. The resulting visualisation indicates that this deceleration begins to emerge after the first few postnatal weeks.

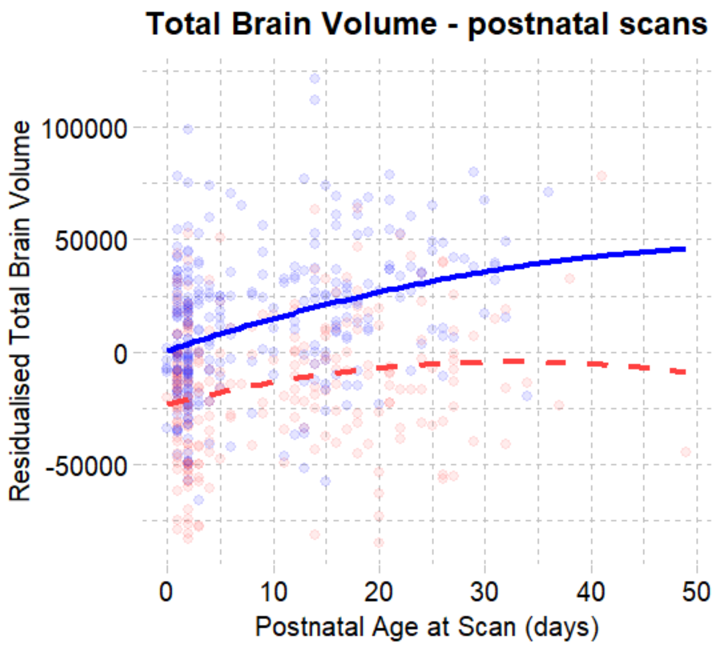

Supplementary Figure 4. Comparison of a quadratic versus logarithmic model for white matter

Brain volumes in mm^3 plotted against postconceptional age in weeks both using a quadratic model and a logarithmic model (measurements transformed back to original units for visualisation).

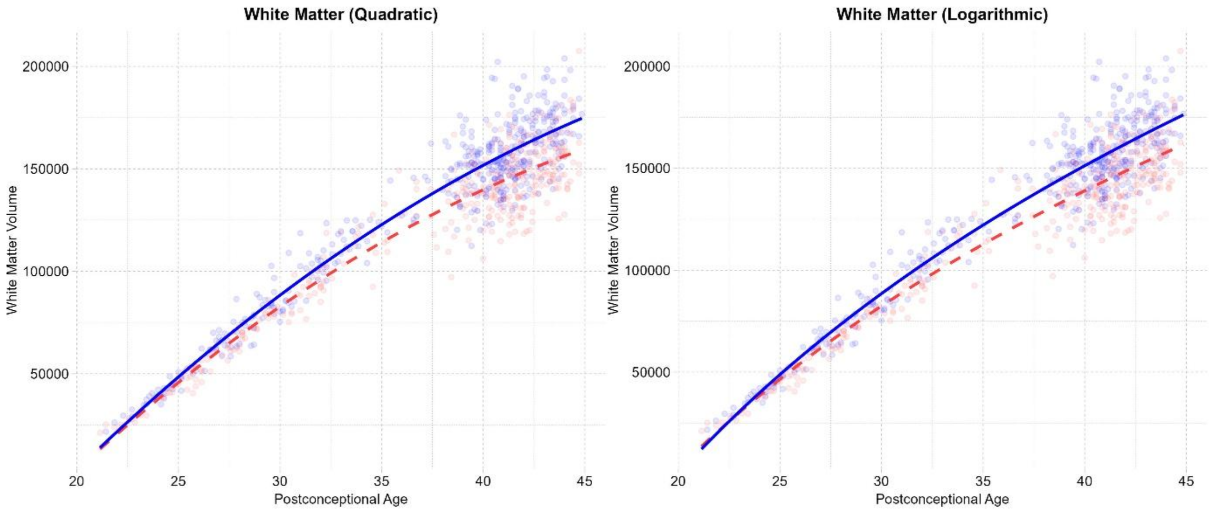

Supplement: Supplementary file 1 — Supplementary Material 1 [file 41598_2025_33981_MOESM1_ESM.pdf]
